# Screening E3 Substrates Using a Live Phage Display Library

**DOI:** 10.1371/journal.pone.0076622

**Published:** 2013-10-04

**Authors:** Zhengguang Guo, Xiaorong Wang, Huihua Li, Youhe Gao

**Affiliations:** 1 Department of Physiology and Pathophysiology, National Key Laboratory of Medical Molecular Biology, Institute of Basic Medical Sciences, Chinese Academy of Medical Sciences/School of Basic Medicine, Peking Union Medical College, Beijing, China; 2 Department of Core Instrument Facility, Institute of Basic Medical Sciences, Chinese Academy of Medical Sciences/School of Basic Medicine, Peking Union Medical College, Beijing, China; 3 Department of Pathology, Institute of Basic Medical Sciences, Chinese Academy of Medical Sciences/School of Basic Medicine, Peking Union Medical College, Beijing, China; Florida International University, United States of America

## Abstract

Ubiquitin ligases (E3s) determine specificity of ubiquitination by recognizing target substrates. However, most of their substrates are unknown. Most known substrates have been identified using distinct approaches in different laboratories. We developed a high-throughput strategy using a live phage display library as E3 substrates in *in vitro* screening. His-ubiquitinated phage, enriched with Ni-beads, could effectively infect *E. coli* for amplification. Sixteen natural potential substrates and many unnatural potential substrates of E3 MDM2 were identified through 4 independent screenings. Some substrates were identified in different independent experiments. Additionally, 10 of 12 selected candidates were ubiquitinated by MDM2 *in vitro*, and 3 novel substrates, DDX42, TP53RK and RPL36a were confirmed *ex vivo*. The whole strategy is rather simple and efficient. Non-degradation substrates can be discovered. This strategy can be extended to any E3s as long as the E3 does not ubiquitinate the empty phage.

## Introduction

Ubiquitination is a key mechanism in regulating many biological processes, such as proteasome degradation, endocytic trafficking, DNA repair, signal transduction and protein-protein interaction [1,2]. In these processes, ubiquitin ligases (E3s) determine the specificity of ubiquitination by recognizing target substrates [1,3-5]. However, most of their substrates that account for the biology are not known [1]. Identification of these substrates is a major challenge. Most substrates have been identified using distinct approaches in different laboratories. Better, faster and cheaper proteome-wide methods are needed to identify E3 substrates [1].

Recently, several powerful methods, including *in vitro* protein microarrays [2-6], *ex vivo* label-free [7] or stable isotope labeling by amino acids in cell culture (SILAC) [8,9] quantitative mass spectrometry and global protein stability (GPS) profiling [10,11] have been employed to identify E3 substrates. *In vitro* protein microarrays have been successfully used to explore substrates of the anaphase promoting complex (APC) [4], SMURF1 [3] and Nedd4 family E3s [2,5,6]. Thousands of candidate proteins were individually expressed, purified, and spotted on arrays. Then, the candidate proteins were incubated with a reaction mixture containing the E3 of interest and FITC-labeled ubiquitin under specific conditions. This is a high-throughput method and can be applied to low amounts of substrate [7] but is limited by the quantity and variety of candidate proteins covered by the arrays [2,5]. As *ex vivo* methods, the label-free and SILAC quantitative mass spectrometry strategies enable the identification of native substrates in physiologically relevant settings [7,8] and have no limitations on the quantity and variety of candidates. Another *ex vivo* method, global protein stability (GPS) profiling of a library of 8000 open reading frames (ORFs) coupled with either a flow cytometry enrichment strategy or a DNA microarray deconvolution strategy was employed to search for SCF substrates in mammalian cells [10,11]. Both the mass spectrometry-based and GPS profiling *ex vivo* methods could only identify the E3 substrates degraded by the proteasome; also, the possibility that candidate proteins decreased following the expression of functional E3 but not due to E3-mediated ubiquitination cannot be excluded. Simpler, more efficient systematic methods are needed to identify E3 substrates.

Phage display is a high-throughput method for the study of protein-protein, protein-peptide, and protein-DNA interactions that uses bacteriophages to connect proteins with the genetic information that encodes them [12]. Phage display has been used in screening enzyme inhibitors and substrates [13]. It was used to determine substrate specificity of proteases [14,15], kinases [16,17], and transglutaminases [18,19]. Here, we developed a screening method using a live phage display library as E3 substrates.

MDM2 was used as an example to explain and evaluate our strategy. The *mdm2* gene was originally identified as one of three genes amplified in tumorigenic mouse cells derived from the NIH3T3 cell line [20,21]. MDM2 acts as an onco-protein that affects the cell cycle, apoptosis, and tumorigenesis through interactions with other proteins or ubiquitination of its substrates. MDM2 is located in many tissues, such as brain, placenta, uterus and lymph node, and is upregulated in many tumor issues [22-24]. It functions as an E3 that mediates ubiquitination of P53/TP53, leading to its proteasome-dependent degradation [25,26]. In addition to P53/TP53, many substrates of MDM2 have been identified individually in different laboratories; these substrates include CDKN1A [27-29], HIPK2 [30,31], RB1 [32-34], CDH1 [35], DLG4 [36], IGF1R [37], APEX1 [38], ADRBK1 [39], ARRB1 [40], ARRB2 [41], CREBBP [42], EID1 [43], IRS1 [40], JMY [44], KAT2B [45], KAT5 [46], MDM4 [47,48], NFATC2 [49], NOL3 [50], RPL26 [51] and itself [52].

## Materials and Methods

### 1. Preparation of plasmids and mutagenesis

For the *in vitro* ubiquitination assay, candidate MDM2 substrates were amplified by PCR from phage clones and subcloned into EcoRI/HindIII sites or EcoRI/XhoI sites of the PET32b+ vector (69016-3, Novagen) (Madison, WI) and fused with His- and S-tags at the N-terminal of the substrates. For the *ex vivo* ubiquitination assay, the full-length human MDM2 (1-491aa) and MDM2Δring (1-435aa) were amplified by PCR from IMAGE: NM_002392.1 (GeneCopoeia Inc.) (Rockville, MD) and subcloned into the BamHI/XhoI sites of the pcDNA6 vector and fused with an N-terminal Flag tag. pEGFP-N1-RPL36AL-GFP, pEGFP-N1-TP5RK-GFP and pEGFP-N1-DDX42-GFP plasmids were all purchased from GeneChem (Shanghai, China), whose gene sequences were obtained from BC000741, BC009727 and BC015505 respectively, and subcloned into the pEGFP-N1 vector and fused with a C-terminal GFP tag. GFP-tagged TP53 plasmid was purchased from GeneCopoeia Inc. (Rockville, MD), whose gene sequence was obtained from IMAGE: NM_000546.2 and subcloned into the pReceiver-M98 vector and fused with a C-terminal eGFP tag. The HA-tagged ubiquitin plasmid and the His-Myc-tagged ubiquitin plasmid were kindly provided by Dr. Huihua Li (Institute of Basic Medical Sciences, Chinese Academy of Medical Sciences).

### 2. Antibodies

All the antibodies used in our study were purchased from commercial sources: anti-S-tag (ab18616, Abcam) (Cambridge, UK), anti-T7-tag (ab9138, Abcam), anti-Flag-tag (DYKDDDDK) (M20008, Abmart) (Shanghai, China), anti-GFP-tag (M20004, Abmart), anti-HA-tag (M20003, Abmart) and anti-β-actin (A1978, Sigma) (St Louis, MO).

### 3. Protein expression and purification

The pET 32b+ constructs were expressed in the *E. coli* BL21 (DE3) strain. The expression of His-tagged proteins was induced by exposure to 0.2 mM IPTG at 30°C for 4-6 hours. The fusion proteins were enriched using MagExtractor(His-tag) (NPK-701, TOYOBO) (Osaka, Japan). The enriched proteins were visualized using SDS PAGE stained with Coomassie Blue and quantified using the Bradford method.

### 4. Ubiquitination screening using live phage as substrate

#### 4.1. Positive and negative selections

A T7Select Human Brain cDNA Library (70637-3, Novagen) was used for MDM2 substrate screening. Approximately 1.7×10^7^ PFU phage was added to the in vitro ubiquitination system containing 110 ng ubiquitin activating enzyme (E1) (UBE1, human recombinant) (E-305, Boston Biochem) (Cambridge, MA), 300-500 ng ubiquitin conjugating enzyme (E2) (UbcH5b, human recombinant) (E2-622, Boston Biochem), 300-830 ng GST-MDM2 (E3-202, Boston Biochem) or GST, 2-3 µg His-ubiquitin (U530, Boston Biochem), 50 mM Tris-HCl (pH=7.4), 7.5 mM MgCl_2_, 3 mM ATP and 1 mM DTT in 20µL volumn. The reaction mixture was incubated at 30°C for 90 min and stopped by the addition of 80 µl Urea Buffer (containing 4 M urea, 10 mM Tris-HCl, 100 mM Na_2_HPO_4_, pH=8.0). Five microliters Ni-agarose beads were added to the reaction mixture and incubated for 1 h at room temperature. For negative selection, the supernatant was collected and amplified in *E. coli*. BLT5403 (69142, Novagen). For positive selection, the supernatant was removed, and the beads were washed with 100 µl Washing Buffer (containing 4 M urea, 10 mM Tris-HCl, 100 mM Na_2_HPO_4_, 1% Triton X-100, pH=8.0) for 5 times. The bound phage was eluted from the beads using 200 mM imidazole for 15 min. The eluted phages were amplified in *E. coli*. BLT5403. The phage titer of the amplified sub-library was determined and put into the in vitro ubiquitination system again for a second round of selection. After 3-5 rounds of selection, the phage titer of the eluent from the last positive selection was determined. Individual clones were selected from the titer plates, amplified by PCR using the T7Select UP and DOWN primers. PCR products were sequenced to obtain the potential E3-substrate coding sequences.

#### 4.2. Trypsin treatment of the ubiquitinated phage

Speculating that phage with a long poly-ubiquitin chain may have a lower *E. coli* infection efficiency. Poly-ubiquitinated phages were digested before infection in the hope that removing the long ubiquitin chain would equalize the infection efficiencies of both poly- and mono-ubiquitinated substrates. The T7 cDNA library was added to the *in vitro* ubiquitination system containing E1, E2, and E3 (MDM2). The reaction product was incubated with Ni-beads, and the beads were washed 5 times with Washing Buffer as described above. The bound phage was treated with 0.125% trypsin (cc035, MACGENE) (Beijing, China) at 37°C for 30 min. After that, the sample was centrifuged and the supernatant was collected. The remaining bound phages were eluted with 200 mM imidazole for 15 min. All the phages were collected from digestion and elution solutions and amplified in BLT5403.

### 5. *In vitro* ubiquitination assay

*In vitro* ubiquitination reactions were performed using 110 ng E1, 500 ng E2, 830 ng E3(GST-MDM2) or GST, 3 μg His-ubiquitin, 50 mM Tris-HCl (PH=7.4), 7.5 mM MgCl2, 3 mM ATP, 1 mM DTT and 100-200 ng substrate in 20µL volume. The reaction mixture was incubated at 30°C for 90 min and stopped by adding 5×SDS sample loading buffer (Genestar Biosolutions, Beijing, China). Proteins were separated by SDS PAGE and immunoblotted with anti-S-tag antibody.

### 6. *Ex vivo* ubiquitination and degradation assays

HEK293T cells, used for the *ex vivo* assays, were cultured under conventional conditions at 37.0°C with 5% carbon dioxide (CO_2_). HEK293T cells were transfected with Flag-tagged MDM2, HA-tagged ubiquitin and GFP-tagged substrates using I’ma Fect Transfection Reagent (IMA201101, IMAGEN). Harvested cells were lysed using RIPA lysis buffer (P1053, Applygen, containing 50 mM Tris-HCl, 150 mM NaCl, 1% NP-40, 0.1% SDS, pH=7.4) (Beijing, China) with 2 mM PMSF and sonication. For each immunoprecipitation, cell lysates were centrifuged at 12000rpm for 10min, and the supernatants were incubated with the appropriate antibodies at 4°C overnight. The protein G-agarose (P2009, Beyotime) (Shanghai, China) was added, allowed to bind the immunocomplex for 1-2 h, and then washed 3 times with cold PBS. Proteins were separated by SDS PAGE and immunoblotted with the appropriate antibodies. In experiments using a proteasome inhibitor, transfected cells were incubated with 10-20 µM MG132 (S1748, Beyotime) for 12 h before harvest.

### 7. Ingenuity pathways analysis (IPA)

IPA software (Ingenuity Systems, Redwood City, CA) was used to investigate possible interactions among all the identified substrates and MDM2. A protein network is a graphic representation of molecular relationship between molecules. Nodes represent molecules, and the lines represent direct and indirect biological relationship. All the lines are supported by at least one reference.

## Results

### 1. E3-substrate screening using a live phage display library

**Figure 1:**
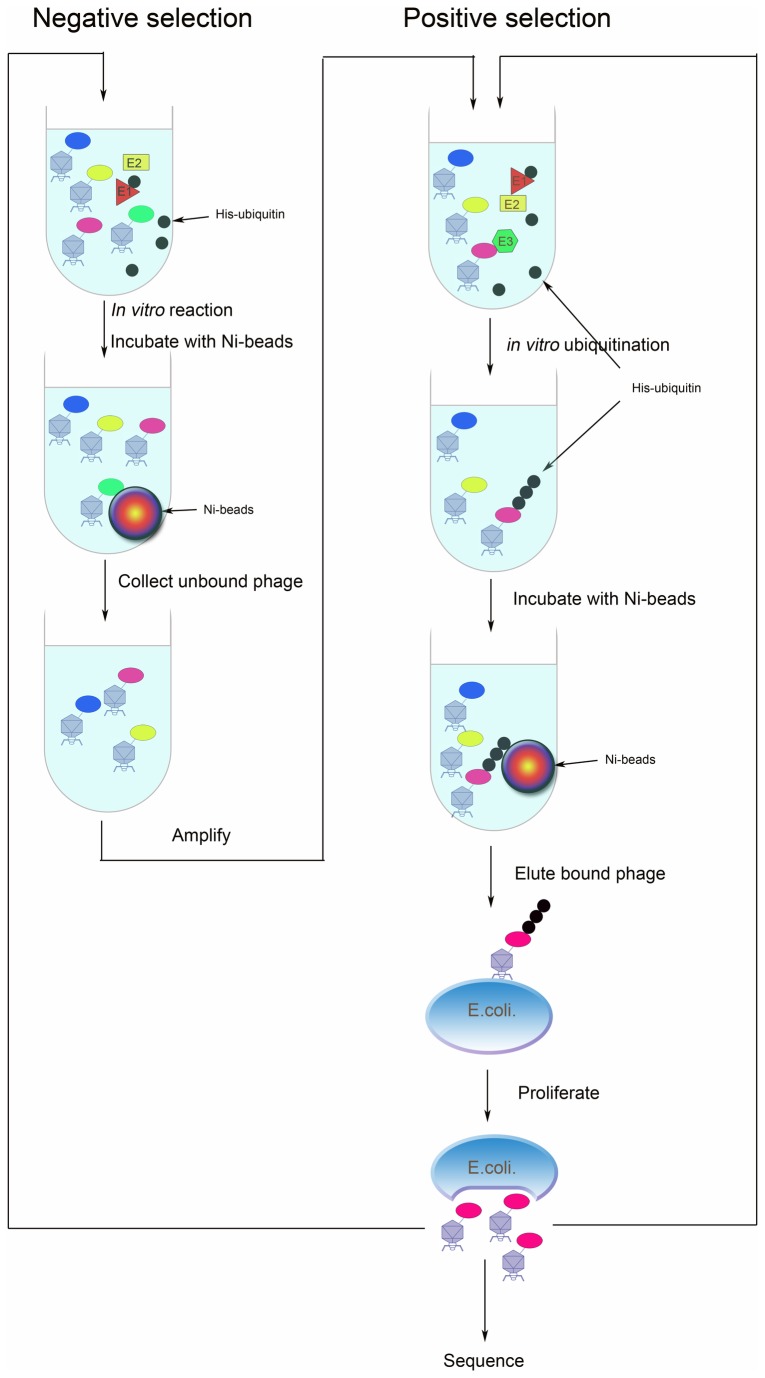
Schematic of the proteomic strategy using a live phage display library as E3 substrates for screening. In negative selection, the library that displayed human brain cDNA was incubated in the *in* *vitro* ubiquitination system containing E1, E2, and His-tagged ubiquitin without E3. The reaction product was incubated with Ni-beads. The unbound phage was collected and amplified in *E*. *coli* BLT5403. The amplified sublibrary was used for positive selection. In positive selection, the sublibrary was incubated in the *in* *vitro* ubiquitination system containing E1, E2, target E3 and His-tagged ubiquitin. The substrates that displayed on phages were mono- or poly-ubiquitinated by the E3 in the system. Then, the reaction products were incubated with Ni-beads. Phages with a His-ubiquitin tag were captured by Ni-beads. The bound phage was further eluted by imidazole and proliferated in BLT5403. The amplified sublibrary from positive selection was put into a second round of positive or negative selection. After several rounds of selection, individual clones were amplified by PCR and sequenced to obtain the potential E3-substrate coding sequences.

The newly developed strategy involves the following steps, as illustrated in Figure **1**.

**First**, to ensure the low background for successful screening in our strategy, E3 should not ubiquitinate the empty phage.

**Second, Negative selection.** Negative selection will eliminate the phage that bound the beads in the system without E3. The library that displayed human brain cDNA was incubated in the *in vitro* ubiquitination system containing E1, E2, and His-tagged ubiquitin without E3. The reaction product was incubated with Ni-beads. The unbound phage was collected and amplified in BLT5403. The amplified sub-library was used for positive selection.

**Third, Positive selection.** The sub-library was incubated in the *in vitro* ubiquitination system containing E1, E2, E3 and His-tagged ubiquitin. The substrates that displayed were mono- or poly-ubiquitinated by the E3 in the system. Then, the reaction products were incubated with Ni-beads. Phages with the His-ubiquitin tag were captured by Ni-beads. The bound phages were further eluted by imidazole and proliferated in BLT5403.

**Fourth, combination of multiple rounds of positive and negative selection.** Multiple rounds of positive selection would further enrich phages that were ubiquitinated by MDM2. The amplified sub-library from positive selection was put into the second round of positive or negative selection. After several rounds of selection, individual clones were PCR amplified and sequenced to obtain the potential E3-substrate coding sequences.

### 2. Screening MDM2 substrates

**Figure 2:**
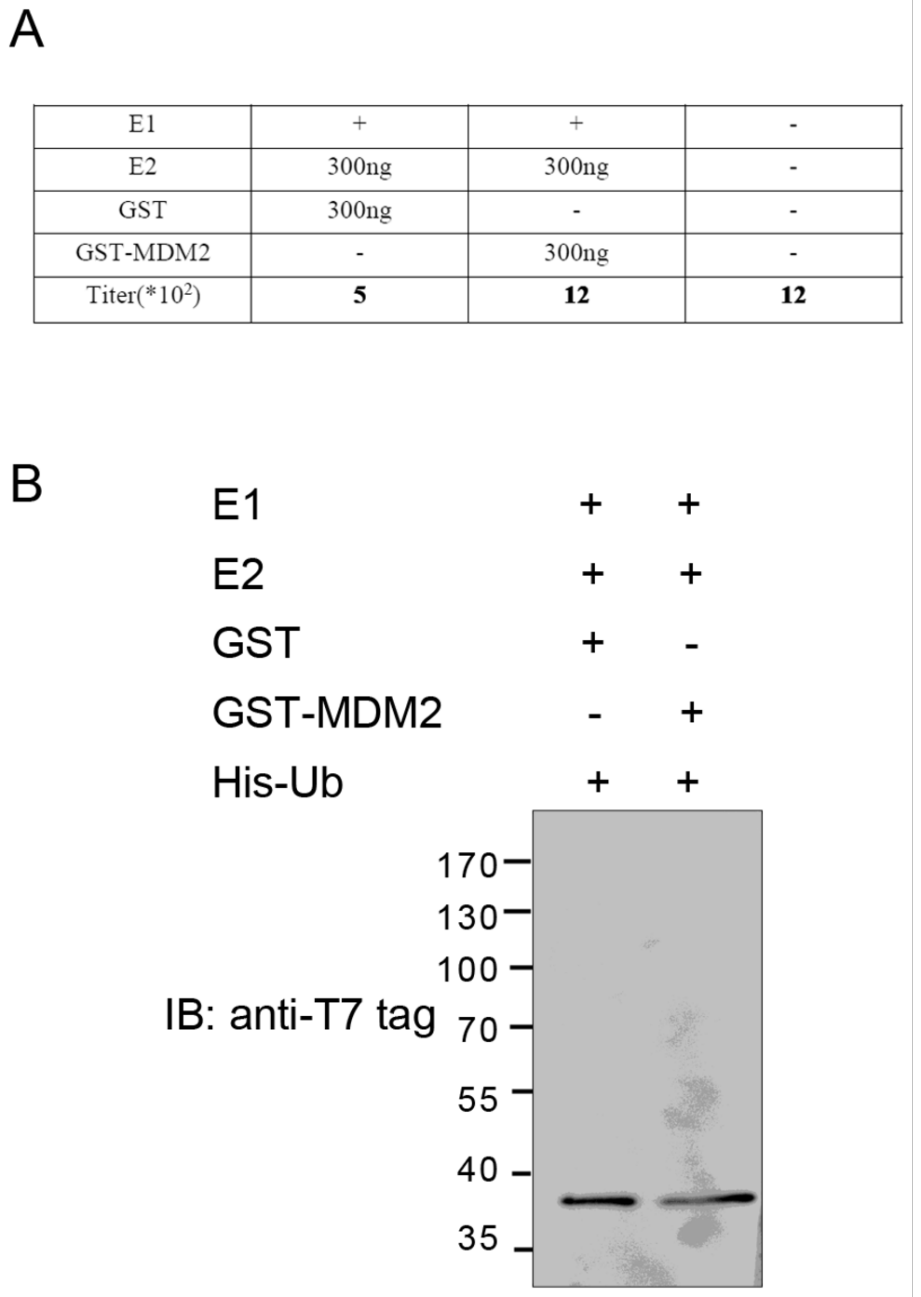
Ubiquitin ligase MDM2 does not ubiquitinate the empty phage. A. Empty phage, after the reaction as substrate in the *in* *vitro* ubiquitination system containing GST-MDM2 as E3, had low nonspecific absorption to the Ni-beads (1.2×10^3^ PFU), as did empty phages not subjected to the reaction (1.2×10^3^ PFU), compared to the input (2×10^7^ PFU). B. MDM2 did not ubiquitinate the coat protein of T7 phage in Western blot analysis.

To test this substrate screening strategy, nonspecific ubiquitination of empty phage (T7Select 10-3, which were kindly provided by Lina Zhang (Beijing Tuberculosis and Thoracic Tumor Research Institute)) by the ubiquitin ligase MDM2 was evaluated. As shown in Figure **2A**, empty phage, when as the substrate in the *in vitro* ubiquitination system containing GST-MDM2 as E3, had low nonspecific absorption to the Ni-beads (1.2×10^3^ PFU), as did empty phages not subjected to the reaction (1.2×10^3^ PFU), compared to the input (2×10^7^ PFU). Western blotting with anti-T7 tag was performed to further validate that the T7-tagged coat protein 10B of the empty phage was not ubiquitinated by MDM2 (Figure **2B**). MDM2 did not ubiquitinate empty phage.

Overall, 10^7^ empty phages and T7 cDNA phage library were added into separate *in vitro* ubiquitination systems; the number of eluted T7 cDNA library phage (26×10^2^ PFU) was a few times higher than that of eluted empty phage (6×10^2^ PFU). Therefore the estimated false positive rate of one round of positive selection without negative selection is 23% (6/26). Negative selection and multiple rounds of positive selection will further reduce false positive rate. Substituting different enzymes for E3 might produce different false positive rates.

Four independent experiments were used to screen MDM2 substrates. The sequences of positive and negative selection in each experiment are described in Table **1** row 2. The *in vitro* ubiquitination system is described in Table **1** rows 3-6. In Experiments 3 and 4, higher concentrations of E1, E2, E3 and His-ubiquitin but fewer rounds of selection were used. In Experiment 4, each positive selection round was followed by trypsin treatment prior to infection, as described above (Table **1** row 7).

**Table 1:** All substrates identified in 4 independent screenings.

		Experiment1	Experiment2	Experiment3	Experiment4	Different clone	Direct association	Other family member	Network	*in vitro* validation	*ex vivo* validation
Selection sequence		**NPPPNPP**	**NPNPNP**	**NPPP**	**NPPP**						
Reaction system	E1	110ng	110ng	110ng	110ng						
	E2	300ng	300ng	**500ng**	**500ng**						
	E3	300ng	300ng	**830ng**	**830ng**						
	His-Ubiquitin	2ug	2ug	**4ug**	**4ug**						
Trypsin treatment		-	-	-	**+**						
Total clones		40	40	83	53						
Natural proteins		22	6	36	28						
NOLC1		**15**	**4**	**5**	**20**	**+**	**+**		**+**	+	NA
DDX42		**1**		**22**		-		**+**	**+**	+	+
MAP2		2				-	**+**		**+**	+	NA
NUCKS1		2				**+**	**+**		**+**	+	NA
RRP1		1							**+**	NA	NA
RBBP6		**1**	**1**			-	**+**	**+**	**+**	+	NA
PDS5			1						**+**	NA	NA
RPL36A				1				**+**	**+**	+	+
UBTF				2		**+**			**+**	NA	NA
TP53RK				1					**+**	+	+
RPL15				**4**	**3**	**+**		**+**	**+**	+	NA
NPIPL3				1					**+**	NA	NA
HMGN1					1				-	-	NA
MAP1LC3A					1				**+**	NA	NA
PRDM2					1			**+**	**+**	-	NA
C12orf35					2	-			**+**	+	NA
Non-natural proteins		18	34	47	25						

Each N or P represents one round of negative or positive selection, respectively; +: positive result; -: negative result; NA: do not tested.

After four independent screenings, 216 clones were obtained. Of these clones, 92 encode proteins with correct ORFs of known proteins, for a total of 16 proteins, as shown in Table **1** column 1. All the raw data are shown in Tables **S1**-**S4** and Text **S1**. NOLC1 was identified in all four experiments. DDX42 was identified in Experiments 1 and 3. RBBP6 was identified in Experiments 1 and 2. RPL15 was identified in Experiments 3 and 4. Four proteins (NOLC1, RPL15, NUCKS1, UBTF) were identified in different clones encoding the same protein (Table **1** column 6). In total, 8 proteins were identified at least twice. These proteins are therefore identified with high-confidence as putative MDM2 substrates. These proteins were validated with *in vitro* and *ex vivo* experiments, discussed in detail later in the results.

**Figure 3:**
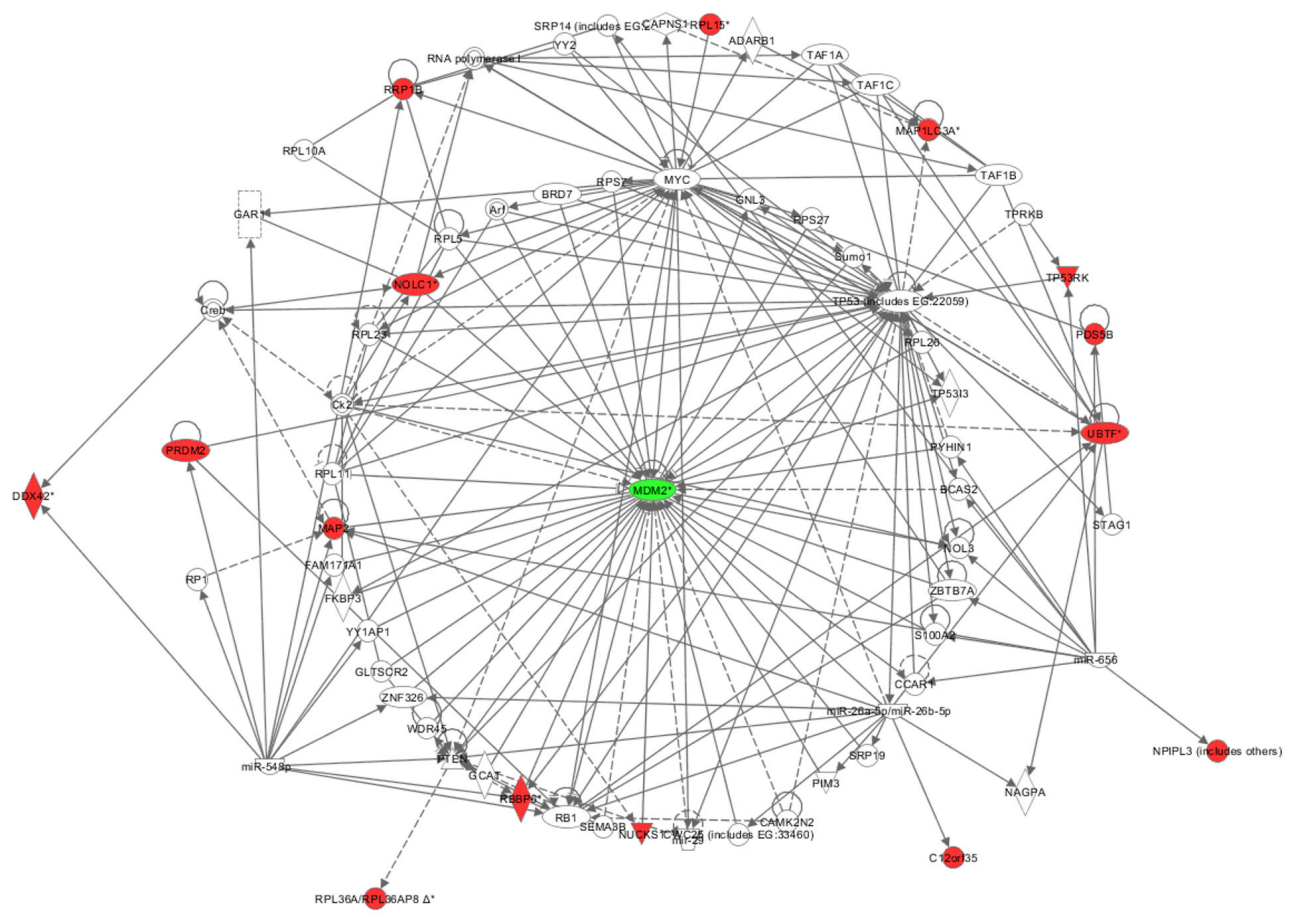
Protein network analysis of MDM2 substrates by Ingenuity Pathway Analysis software (IPA). The genes shaded reds are substrates selected in our strategy. Solid lines represent direct interactions, dotted lines represent indirect interactions. Arrows from one node to another indicate that this node acts upon the other. Lines without arrows represent binding. Node shapes are: vertical diamond means enzyme; dotted rectangle means ion channel; inverted triangle means kinase; horizontal diamond means peptidase; triangle means phosphatase; horizontal oval means transcription regulator; double-circle means complex/group; trapezium means microRNA; semicircle means mature microRNA; circle means other;.

We analyzed the 16 potential MDM2 substrates using IPA software. As shown in Figure **3**, 15 proteins were involved in the MDM2-P53 Network (Table **1** column 9). Of these 15 proteins, 4 have previously been determined to interact or have a functional association with MDM2 by independent laboratories (Table **1** column 7). Endogenous PACT (RBBP6) [53] can interact with MDM2 and enhance MDM2-mediated ubiquitination and degradation of P53, and increase the P53-MDM2 affinity. Endogenous NOLC1 [54] interacts with the promoter of MDM2 together with P53 to activate the MDM2 promoter in NPC cells. A large-scale protein interaction study using an mRNA display showed NUSCK1 and MAP2 interact with MDM2 [55]. Another 11 proteins, which were indirectly associated with MDM2, also existed in the MDM2-P53 network. For example, PRDM2 interacts with P53 and regulates the proliferation of monocytic leukemia cells via activation of P53 [56]. PRDM2 also interacts with Rb1, another MDM2 substrate [57,58]. P53 posttranscriptionally down-regulates autophagy protein LC3 (MAP1LC3), supporting cancer cell survival under prolonged starvation [59]. Another large scale protein interaction study by mass spectrometry discovered that PDS5B interacts with MYC, a significant MDM2 regulator [60]. TP53RK binds to TP53, and phosphorylates P53 [TP53] protein to phosphorylated (S15) P53 [TP53] protein [61]. Mutant RB1 (C706F) interacts with and decreases DNA binding activity of human UBF1 (UBTF) protein [62-64]. P53 prevents the interaction between SL1 and UBTF, and represses RNA Pol I transcription activity [65]. Chromatin-associated factor RRP1B interacts with RPL5, a MDM2 binding protein [66]. An important MDM2 regulator, PTEN increases expression of mouse RPL44 [RPL36a] mRNA in mouse mammary gland tissue [67]. The family members of 4 proteins are known physiological substrates or interactors of MDM2 (Table **1** column 8). RPL36a and RPL15 are members of the RPL family. As members of this family, L5 [68], L11 [69] and L23 [70,71] interact with MDM2 to inhibit MDM2-mediated P53 ubiquitination. Another member of this family, L26 [51], binds to MDM2, which leads to its polyubiquitinylation and proteasomal degradation. DDX42 is a member of the DEAD box (*DDX*) family, one member of which, DDX17 [72], combines with p300 and P/CAF to stimulate MDM2 promoters. PRDM2 is a member of the PRDM (PRDI-BF1 and RIZ domain containing) family, one member of which, PRDM5 [73], down-regulates MDM2 gene expression to inhibit tumor cell clonogenicity and cell proliferation.

The remaining 124 clones do not encode natural proteins with correct ORFs. Some clones match the genome sequence but not the cDNA sequence, some match the non-coding region of the cDNA, some match reverse cDNA sequences, some have frameshifts, and others match neither the genome nor the cDNA sequences. These results indicated that the library also displayed other peptides. Twelve polypeptides were identified at least twice in the 124 clones, as shown in Table **S5**. One was identified in different independent experiments, and at least six were identified in different clones that encode the same peptides. These peptides are high-confidence unnatural substrates of MDM2. Interestingly, many clones contain poly-lysine, which likely occurred for several reasons. MDM2 has Asp/Glu-rich (acidic residue-rich) region at 243-301 aa and might interact with basic residue-rich poly-lysine peptides and cause its ubiquitination. The enrichment in the cDNA library of the AAA codon derived from the poly-A region of mRNA, which encodes lysine, may be the cause of poly-lysine peptides in the library.

### 3. *In vitro* validation of MDM2 substrates

One potential caveat of this strategy is that the N-terminal phage coat protein fusion might affect protein folding and ubiquitination. To validate that the identified substrates are ubiquitinated by MDM2, 11 natural novel potential substrates and 1 unnatural potential substrates of MDM2 were selected from the phage display screening and inserted into the bacterial expression plasmid PET32b+. The empty vector was used as a negative control. Each recombinant protein was fused with a tandem His and S tag at the N-terminus for purification and detection. Each recombinant protein was added to the *in vitro* ubiquitination reaction containing E1, E2 (UbcH5B), E3 (GST-MDM2 or GST) and ubiquitin. Ubiquitination of the potential substrates was detected by anti-S tag antibody.

**Figure 4:**
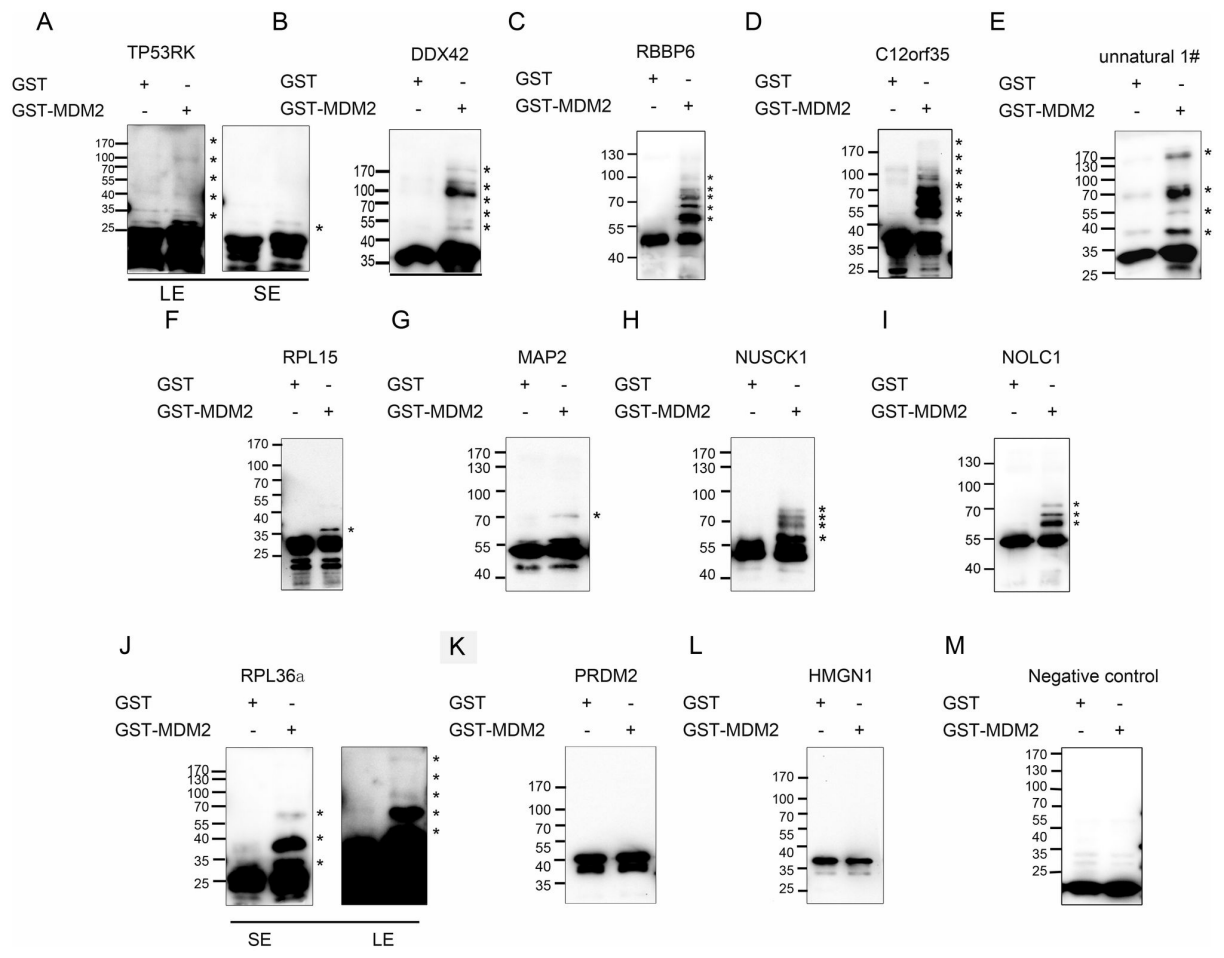
*In vitro* validation of candidate MDM2 substrates selected by the phage display strategy. Candidate substrates TP53RK (A), DDX42 (B), RBBP6 (C), C12orf35 (D), the unnatural substrate 1# (E), RPL15 (F), MAP2 (G), NUSCK1 (H), NOLC1 (I), RPL36a (J), PRDM2 (K), HMGN1 (L) and the negative control (M) were purified and subjected to traditional liquid ubiquitination reactions. They were then separated by SDS PAGE and immunoblotted with an anti-S tag antibody. All candidate substrates were ubiquitinated by MDM2, except PRDM2 (K) and HMGN1 (L). *: ubiquitination bands. SE: short exposure. LE: long exposure.

As shown in Figure **4** and summarized in Table **1** column 10, 10 of the 12 potential substrates identified in the phage display screening were ubiquitinated by the ubiquitin ligase. Among them, RPL15a was mono-ubiquitinated by MDM2 in the *in vitro* ubiquitination assay. MAP2 was bi-ubiquitinated by MDM2 in the *in vitro* ubiquitination assay. NOLC1, NUSCK1 and RPL36a were mainly oligo-ubiquitinated by MDM2. TP53RK, DDX42, C12orf35, RBBP6, and the unnatural potential substrate were poly-ubiquitinated by GST-MDM2 compared to GST in solution. However, the ubiquitination of the PRDM2 and HMGN1 was not detected. The protein expressed by the empty vector of PET32b+, used as a negative control, was not ubiquitinated by MDM2.

### 4. *Ex vivo* confirmation of MDM2 substrates

**Figure 5:**
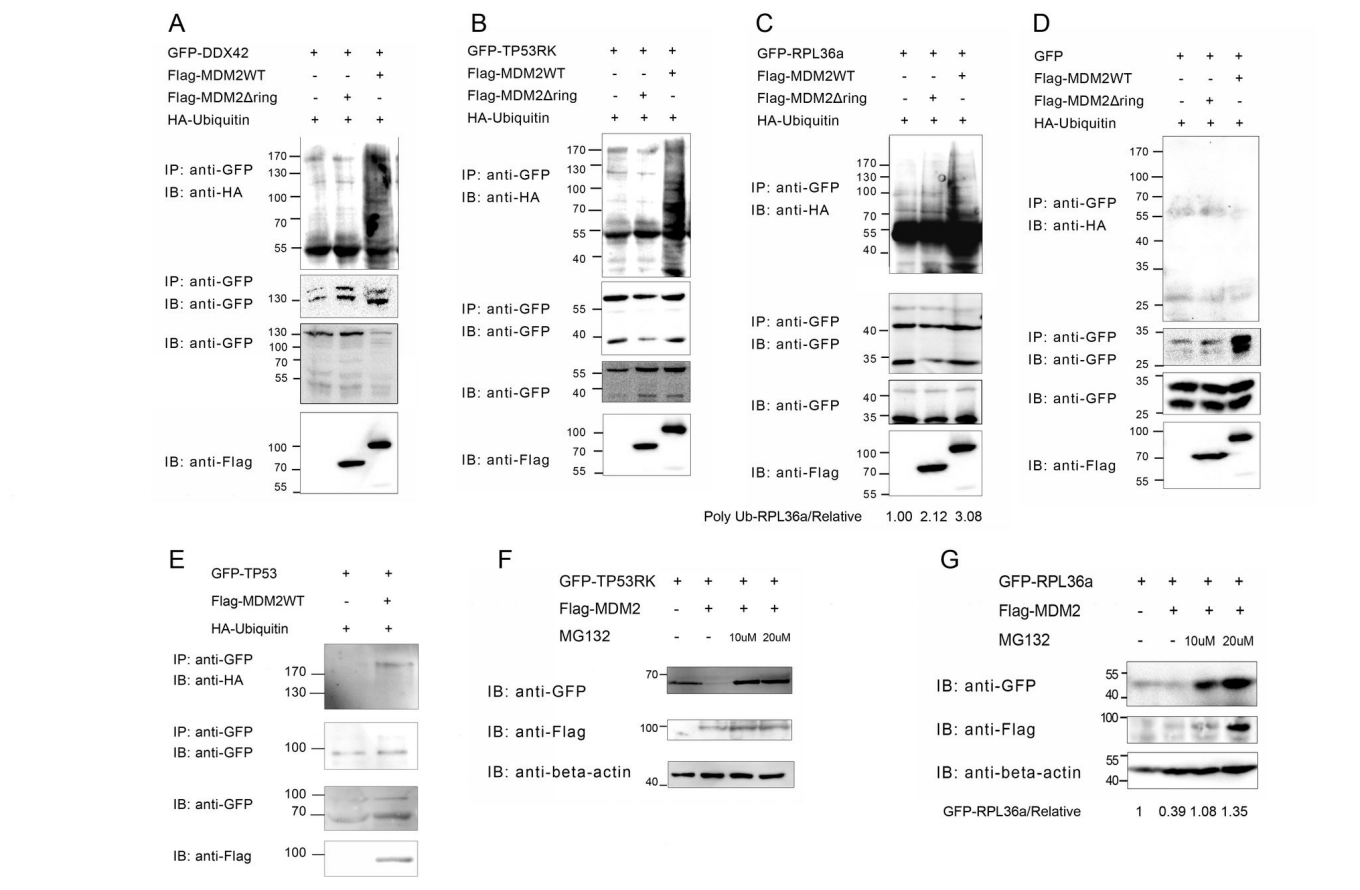
Confirmation of MDM2 substrates in mammalian cells by an *ex vivo* ubiquitination assay. HEK293T cells were transfected with the GFP-tagged substrates alone or co-transfected with Flag-tagged MDM2 (either WT or a catalytically inactive MDM2Δring mutant). The GFP-tagged substrate was immunoprecipitated from cell lysates with the anti-GFP antibody and immunoblotted with the anti-HA antibody to detect its ubiquitination. DDX42 (A) and TP53RK (B) were ubiquitinated when MDM2 was co-expressed. TP53RK and DDX42 were ubiquitinated when MDM2 was co-expressed, whereas only very faint ubiquitination bands were observed in the MDM2Δring lane. RPL36a (C) was ubiquitinated when MDM2 was co-expressed, while weaker ubiquitination bands were observed in the MDM2Δring lane. To calculate the relative extent of RPL36a poly-ubiquitination, the poly-ubiquitination signal of RPL36a (poly-Ub-RPL36a) was quantified using ImageJ software, divided by the corresponding GFP-RPL36a signal and normalized to 1.0. HEK293T (D) cells were transfected with empty vector of GV142 expressed only GFP protein (negative control) alone or with co-transfected Flag-tagged MDM2 (either WT or a catalytically inactive MDM2Δring mutant). The GFP was immunoprecipitated from cell lysates with the anti-GFP antibody and immunoblotted with the anti-HA antibody to detect its ubiquitination. GFP itself was not ubiquitinated by MDM2. HA-ubiquitin (E) and GFP-P53 (positive control) were co-transfected into HEK293T cells. The GFP-P53 was immunoprecipitated from cell lysates with the anti-GFP antibody and immunoblotted with the anti-HA antibody to detect its ubiquitination. P53 was ubiquitinated when MDM2 was co-expressed. GFP tagged TP53RK (F) or RPL36a (G) were transfected into HEK293T cells alone or co-transfected with Flag-tagged MDM2. The protein content of the lysates of the transfected cells were separated and immunoblotted using the anti-GFP antibody. The expression of MDM2 induced the significant degradation of transfected TP53RK and RPL36a. When the transfected cells were treated with 10 or 20 μM MG132, the degradation of TP53RK and RPL36a were blocked. *: ubiquitination bands.

Three novel potential substrates of MDM2, including DDX42, RPL36a, and TP53RK, were further tested in *ex vivo* ubiquitination assays. In the *ex vivo* ubiquitination assay, Flag-tagged MDM2 and MDM2Δring were transfected into HEK293T cells with GFP-tagged putative substrates and HA-tagged ubiquitin. MDM2Δring is a truncated form of MDM2 without E3 activity. Putative full-length substrates were immunoprecipitated from cell lysates with anti-GFP antibody and immunoblotted with anti-HA antibody to detect their ubiquitination. As shown in Figure **5** and summarized in Table **1** column 11, in the presence of Flag-MDM2, DDX42 (Figure **5A**) and TP53RK (Figure **5B**) were significantly ubiquitinated by MDM2 compared to Flag-MDM2 non-overexpressed cells, as indicated by the high-molecular-weight ladders and smear, while very faint ubiquitination bands were observed when MDM2Δring was over-expressed. RPL36a (Figure **5C**) was ubiquitinated when MDM2 was co-expressed, while weaker ubiquitination bands were also observed in the MDM2Δring lane. In fact, RPL36a poly-ubiquitination increased by more than 2 fold in MDM2 over-expressed cells, while only 1 fold increase was observed in MDM2Δring over expressed cells (the raw quantification data was shown in Table **S6**). Empty vector of GV142 expressed only GFP protein, which was used as a negative control, was not ubiquitinated by MDM2, indicating that the GFP tag was not ubiquitinated (Figure **5D**); P53, a well-known substrate of MDM2, which was used as a positive control, was significantly ubiquitinated by MDM2 (Figure **5E**).

The ubiquitination of cellular proteins often leads to their proteasome-dependent degradation. We evaluated whether MDM2-induced ubiquitination causes the degradation of DDX42, RPL36a and TP53RK. GFP-tagged DDX42, RPL36a and TP53RK were co-transfected into HEK293T cells with Flag-tagged MDM2. Proteins in the lysates of transfected cells were separated and immunoblotted with anti-GFP antibody. As shown in Figure **5**, the over-expression of MDM2 induced the significant degradation of GFP-tagged TP53RK (Figure **5F**) and RPL36a (Figure **5G**) (the raw quantification data was shown in Table **S7**), while degradation was blocked when treated with 10 or 20 µM MG132 for 12 h. Therefore, MDM2 promoted the degradation of TP53RK and RPL36a in a proteasome-dependent manner. However, the degradation of DDX42 mediated by MDM2 was not detectable (Figure **S1**). This finding suggested that the ubiquitination of DDX42 does not lead to its degradation. The ubiquitination of DDX42 might serve as a nonproteolytic signal in cellular processes.

## Discussion

This strategy is notably different from other approaches in the following respects. First, the whole strategy is simple and efficient. The expression and purification of numerous candidate proteins were unnecessary; the process of phage display screening was very simple and not time-consuming. The whole selection process will be finished less than two weeks, and the cost for sequencing DNA of phage clones is much lower than protein identification. Second, the low false-positive rate (2 of 12) of the screen is favorable compared with those of most high through-put microarray-based approaches [2,11]. Third, not only poly-ubiquitinated and degradation substrates but also the mono-/oligo-ubiquitinated and non-degradation substrates of the target E3s can be discovered. Assembly of a chain of at least four ubiquitins linked together via their Lys48 residue leads to the 26S proteasome-dependent degradation of targeted cellular proteins; in contrast, mono-ubiquitination or poly-ubiquitination with chains linked together via Lys63 serve as nonproteolytic signals in intracellular trafficking, DNA repair, and signal transduction pathways [1]. Cell-surface transmembrane molecules are often mono-ubiquitinated for endocytosis [74]. Histones are usually mono-ubiquitinated and associated with signaling or structural marking [75]. Mono-ubiquitination is also involved in virus budding [74]. K63 linkage of ubiquitin is known to be involved in DNA damage recognition of DNA double-strand breaks. These types of protein modification are important but cannot be easily detected by *ex vivo* high-throughput methods, such as label-free [7] or SILAC [8,9] quantitative mass spectrometry and GPS profiling [10,11] because of the non-degradation fate of the substrates. Additionally, in our strategy, more mono-ubiquitination or oligo-ubiquitination substrates of MDM2 were found. This phenomenon might because the mono- or oligo-ubiquitination modified phages might have higher amplification efficiency in host cells and these clones would have more chance to be identified. We also found that poly-ubiquitination of DDX42 by MDM2 does not cause its degradation.

Previous studies have identified 23 proteins as *bona fide* substrates of MDM2. However, most of these substrates were not identified using our strategy. This discrepancy likely occurred for several reasons. Some of these substrates (Cadherin-1，Dlg4，IGF1R) are membrane proteins, which are difficult to display on the surface of phages. Some of them are very low abundance proteins, which were not included in the library we used. Some of the substrates might require post-translational modifications, which would be lacking in the *in vitro* ubiquitination system, to be recognized by MDM2. Lastly, it has been noticed in previous studies that only a fraction of MDM2 substrates are ubiquitinated in any given *in vitro* system, so several independent selections per target E3 may be necessary to improve the coverage of substrates.

The vector of T7 cDNA library was T7 select 10-3, which could theoretically display up to 1200aa protein. The range of molecular weights of identified natural substrates was from 57aa to 342aa. Many of them were truncated proteins but not full-length proteins. This strategy was suitable to screen substrates of small proteins or truncated large proteins, which could be further validated in the full-length form.

Four different conditions were used to screen the MDM2 substrates: low enzyme concentration and more rounds of positive selection (Experiment 1), more rounds of negative selection (Experiment 2), high enzyme concentration and fewer rounds of selection (Experiment 3), and trypsin treatment during selection (Experiment 4). Some substrates were identified in different independent experiments, and some substrates were identified in only one experiment. Many substrates were identified in both Experiments 1 and 3. Therefore, both Experiment 1 and Experiment 3 were effective, suggesting that the enzyme concentration and rounds of selection were variable in our strategy. Only a few substrates were identified in Experiment 2, which might due to the loss of substrates in excessive rounds of negative selection. We speculated that phage with a long poly-ubiquitin chain may have a lower *E. coli* infection efficiency. Poly-ubiquitinated phages were digested by 0.125% trypsin treatment at 37°C for 30 min in the hope that removing the long ubiquitin chain would equalize the infection efficiencies of both poly- and mono-ubiquitinated substrates in Experiment 4. Theoretically, more poly-ubiquitinated substrates of MDM2 should be identified in Experiment 4. In fact, only one novel poly-ubiquitinated substrate (C12orf35) and many mono- and oligo-ubiquitinated substrates (RPL15 and NOLC1) were identified, indicating that the trypsin treatment did not improve the identification of poly-ubiquitinated substrates. The ubiquitination of HMGN1 and PRDM2 were not detected in the *in vitro* ubiquitination system, indicating that trypsin treatment might introduce false-positive results. Overall, we suggested that more flexible screening conditions could be used. More importantly, several independent selections should be performed for target E3s to expand the coverage of substrates. The results generated from independent experiments are more reliable. High-quality phage libraries will also help us to identify more natural substrates of E3s in our strategy.

Many unnatural substrates were identified in the study. These unnatural substrates could be used to find the consensus of substrates, which might help us to characterize the substrates recognition mechanism. Additionally, non-natural MDM2 substrates might have the potential role to be the competitive inhibitor of MDM2.

This strategy can be extended to substrate discovery for any E3s, as long as the target E3 does not ubiquitinate the empty phage and the E3-substrate recognition does not depend on cellular post-translational modifications which are unfit for the *in vitro* ubiquitination system. There are a series of ubiquitin-like proteins (UBLs), such as SUMOs and NEDD8, involved in post-translational protein modification. The mechanisms of SUMOylation and NEDDylation are similar to that of ubiquitin and are also mediated by corresponding enzymes (E1, E2, E3) [76]. SUMOylation and NEDDylation of target proteins have been successfully characterized by *in vitro* systems. Therefore, our strategy can also be potentially expanded to identify the targets of SUMOylation and NEDDylation. Live phage display libraries could be used as substrates for any protein modification enzyme screening, as long as the enzyme does not modify the empty phage, the modification to the phage does not affect its infection of host cells, and the modified phage can be purified.

## Supporting Information

Figure S1 **MDM2 did not induce the significant degradation of transfected DDX42.** GFP tagged DDX42 was transfected into HEK293T cells alone or co-transfected with Flag-tagged MDM2. The protein content of the lysates of the transfected cells were separated and immunoblotted using the anti-GFP antibody. The expression of MDM2 did not induce the significant degradation of transfected DDX42.
(DOC)

Table S1 **Encoding sequence and encoding protein of clones selected in Experiment 1.**
(DOC)

Table S2 **Encoding sequence and encoding protein of clones selected in Experiment 2.**
(DOC)

Table S3 **Encoding sequence and encoding protein of clones selected in Experiment 3.**
(DOC)

Table S4 **Encoding sequence and encoding protein of clones selected in Experiment 4.**
(DOC)

Table S5 **Unnatural peptides identified at least twice in four independent screenings.** +: positive result; -: nagetive result; NA: do not tested.
(DOC)

Table S6 **Quantification of exogenous ubiquitinated RPL36a using ImageJ in MDM2 overexpressed HEK293T cells.**
(DOC)

Table S7 **Quantification of exogenous RPL36a using ImageJ in MDM2 overexpressed HEK293T cells.**
(DOC)

Text S1 **Raw DNA sequencing results.**
(TXT)
