# Supplementary material for: Screening E3 Substrates Using a Live Phage Display Library
Source: PLoS One. 2013 Oct 4;8(10):e76622. doi: 10.1371/journal.pone.0076622 (PMC3790729; doi:10.1371/journal.pone.0076622)
Supplement: Figure S1 — MDM2 did not induce the significant degradation of transfected DDX42. GFP tagged DDX42 was transfected into HEK293T cells alone or co-transfected with Flag-tagged MDM2. The protein content of the lysates of the transfected cells were separated and immunoblotted using the anti-GFP antibody. The expression of MDM2 did not induce the significant degradation of transfected DDX42. (DOC) [file pone.0076622.s001.doc]

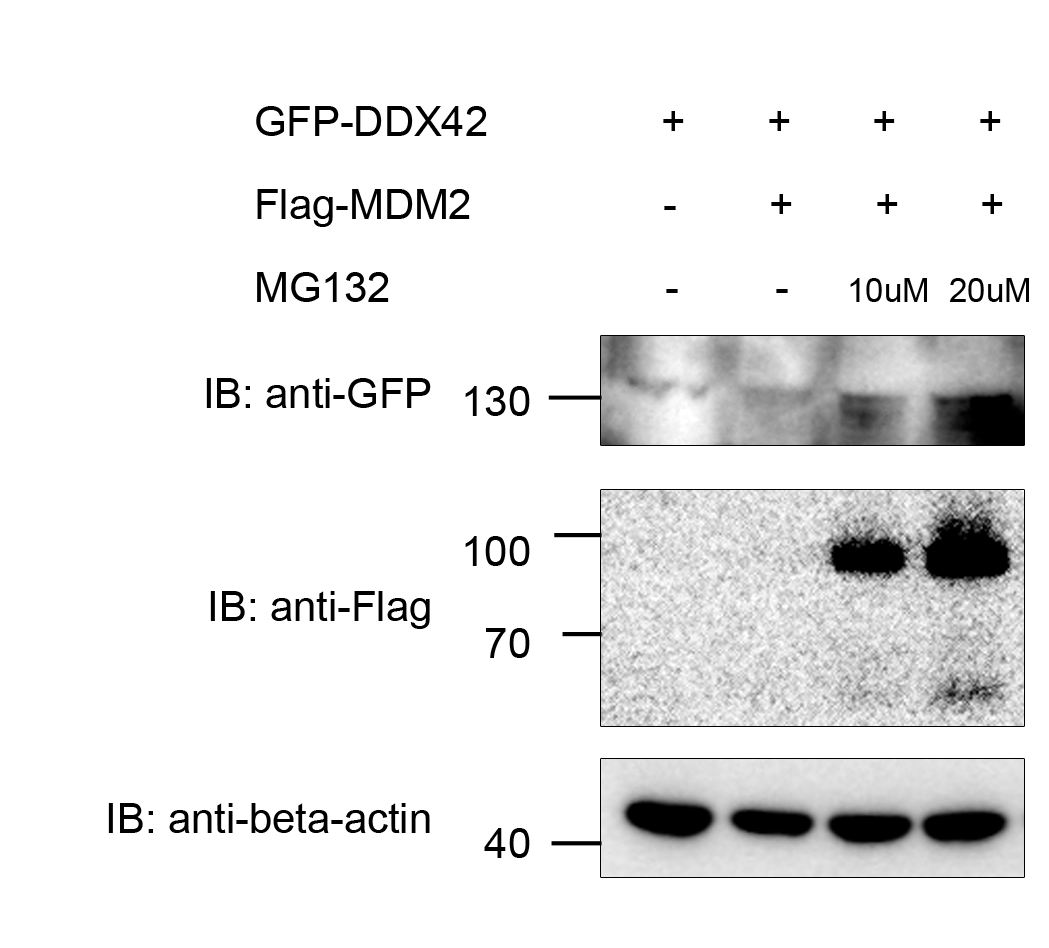


**Figure S1：**MDM2 did not induce the significant degradation of transfected DDX42. GFP tagged DDX42 was transfected into HEK293T cells alone or co-transfected with Flag-tagged MDM2. The protein content of the lysates of the transfected cells were separated and immunoblotted using the anti-GFP antibody. The expression of MDM2 did not induce the significant degradation of transfected DDX42.
