# Supplementary material for: Screening E3 Substrates Using a Live Phage Display Library
Source: PLoS One. 2013 Oct 4;8(10):e76622. doi: 10.1371/journal.pone.0076622 (PMC3790729; doi:10.1371/journal.pone.0076622)
Supplement: Table S1 — Encoding sequence and encoding protein of clones selected in Experiment 1. (DOC) [file pone.0076622.s002.doc]

Table S1: Encoding sequence and encoding protein of clones selected in Experiment 1

| Serial number | Encoding peptide sequence | Encoding protein |
| --- | --- | --- |
| 1 | NSSNKPAVTTKSPAVKPAAAPKQPVGGGQKLLTRKADSSSSEEESSSSEEEKTKKMVATTKPKATAKAALSLPAKQAPQGSRDSSSDSDSSSSEEEEEKTSKSAVKKKPQKVAGGAAPSKPASAKKGKAESSNSSSSDDSSEEEEEKLKGKGSPRPQAPKANGTSALTAQNGKAAKNSEEEEEEKKKAAVVVSKSGSLKKRKQNEAAKEAETPQAKKIKLAAALE- | Homo sapiens nucleolar and coiled-body phosphoprotein 1 (NOLC1), |
| 2 | NSACSRPQETVLCGELIWSVSRTFPANLT- | frameshift |
| 3 | NSSDESNKETANLQERSISNDDGEEKNSNKCASERKKTQTFSHCIR- | frameshift |
| 4 | NSSDESNKETANLQERSISNDDGEEKNSNKCASERKKTQTFSHCIR- | frameshift |
| 5 | NSSDESNKETANLQERSISNDDGEEKNSNKCASERKKTQTFSHCIR- | frameshift |
| 6 | NSSNKPAVTTKSPAVKPAAAPKQPVGGGQKLLTRKADSSSSEEESSSSEEEKTKKMVATTKPKATAKAALSLPAKQAPQGSRDSSSDSDSSSSEEEEEKTSKSAVKKKPQKVAGGAAPSKPASAKKGKAESSNSSSSDDSSEEEEEKLKGKGSPRPQAPKANGTSALTAQNGKAAKNSEEEEEEKKKAAVVVSKSGSLKKRKQNEAAKEAETPQAKKIKLAAALE- | Homo sapiens nucleolar and coiled-body phosphoprotein 1 (NOLC1), |
| 7 | NSSKKKKKKKEKKALQLGRKKK- | No match |
| 8 | NSHSRVSV- | No match |
| 9 | NSVPTNSAQQGHNSPDSPVTSAAKGIPGFGNTGNISGAPVTYPSAGAQGVNNTASGNNSREGTGGSNGKRERYTENRGSSRHSHGETGNRHSDSPRHGDGGRHGDGYRHPESSSRHTDGHRHGENRHGGSAGRHGENRGANDGRNGESRKEACGRTRVTS- | Homo sapiens DEAD (Asp-Glu-Ala-Asp) box polypeptide 42 (DDX42), |
| 10 | NSSDESNKETANLQERSISNDDGEEKNSNKCASERKKTQTFSHCIR- | frameshift |
| 11 | NSSA- | frameshift |
| 12 | NSSKARDITLPNFKLCYKATVTQTA- | genome sequence |
| 13 | NSSNKPAVTTKSPAVKPAAAPKQPVGGGQKLLTRKADSSSSEEESSSSEEEKTKKMVATTKPKATAKAALSLPAKQAPQGSRDSSSDSDSSSSEEEEEKTSKSAVKKKPQKVAGGAAPSKPASAKKGKAESSNSSSSDDSSEEEEEKLKGKGSPRPQAPKANGTSALTAQNGKAAKNSEEEEEEKKKAAVVVSKSGSLKKRKQNEAAKEAETPQAKKIKLAAALE- | Homo sapiens nucleolar and coiled-body phosphoprotein 1 (NOLC1), |
| 14 | NSSNKPAVTTKSPAVKPAAAPKQPVGGGQKLLTRKADSSSSEEESSSSEEEKTKKMVATTKPKATAKAALSLPAKQAPQGSRDSSSDSDSSSSEEEEEKTSKSAVKKKPQKVAGGAAPSKPASAKKGKAESSNSSSSDDSSEEEEEKLKGKGSPRPQAPKANGTSALTAQNGKAAKNSEEEEEEKKKAAVVVSKSGSLKKRKQNEAAKEAETPQAKKIKLAAALE- | Homo sapiens nucleolar and coiled-body phosphoprotein 1 (NOLC1), |
| 15 | NSSGRKRERKRGKEGRREQNRPGAVAQASNLSTLEGQGGRIMRSGDRGHSGHQSETPYLLKIQKLAALGSACL- | no match |
| 16 | NSSNKPAVTTKSPAVKPAAAPKQPVGGGQKLLTRKADRSSSEEESSSSEEEKTKKMVATTKPKATAKAALSLPAKQAPQGSRDSSSDSDSSSSEEEEEKTSKSAVKKKPQKVAGGAAPSKPASAKKGKAESSNSSSSDDSSEEEEEKLKGKGSPRPQAPKANGTPALTAQNGKAAKNSEEEEEEKKKAAVVVSKSGSLKKRKQNEAAKEAETPQAKKIKLAAALE- | Homo sapiens nucleolar and coiled-body phosphoprotein 1 (NOLC1), |
| 17 | NSSNKPAVTTKSPAVKPAAAPKQPVGGGQKLLTRKADRSSSEEESSSSEEEKTKKMVATTKPKATAKAALSLPAKQAPQGSRDSSSDSDSSSSEEEEEKTSKSAVKKKPQKVAGGAAPSKPASAKKGKAESSNSSSSDDSSEEEEEKLKGKGSPRPQAPKANGTPALTAQNGKAAKNSEEEEEEKKKAAVVVSKSGSLKKRKQNEAAKEAETPQAKKIKLAAALE- | Homo sapiens nucleolar and coiled-body phosphoprotein 1 (NOLC1), |
| 18 | NSSNKPAVTTKSPAVKPAAAPKQPVGGGQKLLTRKADRSSSEEESSSSEEEKTKKMVATTKPKATAKAALSLPAKQAPQGSRDSSSDSDSSSSEEEEEKTSKSAVKKKPQKVAGGAAPSKPASAKKGKAESSNSSSSDDSSEEEEEKLKGKGSPRPQAPKANGTPALTAQNGKAAKNSEEEEEEKKKAAVVVSKSGSLKKRKQNEAAKEAETPQAKKIKLAAALE- | Homo sapiens nucleolar and coiled-body phosphoprotein 1 (NOLC1), |
| 19 | NSKYGLQGNPTKSKLKPNTKKQTNKKTTQGMKDEIIILRKKPRRTPATEKFTKGARCGG-/NSNMDCKETQRNPS- | genome sequence |
| 20 | NSPFTRGRREDYVGGQSHRSRNIGSNYPEKLSARDGHNQKDNTKSKEKESENAPGDGKGNKHKKHRKRRKGEESEGFLNPELLETSRKSREPTGVEENKTDSLFVLPSRDDATPVRDEPMDAESITFKSVSEKDKRERDKPKAKGDKTKRKNDGSAVSKKENIVKPAKGPQEKVDGERERSPRSEACGRTRVTS- | Homo sapiens retinoblastoma binding protein 6 (RBBP6), transcript |
| 23 | NSSRRPSPHDEEEFEVEEAAEAQAEPKDGSPEAPASPEREEVALSEYKTETYDDYKDETTIDDSIMDADSLWVDTQDDDRSIMTEQLETIPKEEKAEKEARRSSLEKHRKEKPFKTGRGRISTPERKVAKKEPSTVSRDEVRRKKLAAALE- | Homo sapiens microtubule-associated protein 2 (MAP2) |
| 25 | NSSISHCNHQIPLYDSV- | frameshifts |
| 27 | NSSNKPAVTTKSPAVKPAAAPKQPVGGGQKLLTRKADSSSSEEESSSSEEEKTKKMVATTKPKATAKAALSLPAKQAPQGSRDSSSDSDSSSSEEEEEKTSKSAVKKKPQKVAGGAAPSKPASAKKGKAESSNSSSSDDSSEEEEEKLKGKGSPRPQAPKANGTSALTAQNGKAAKNSEEEEEEKKKAAVVVSKSGSLKKRKQNEAAKEAETPQAKKIKLAAALE- | Homo sapiens nucleolar and coiled-body phosphoprotein 1 (NOLC1) |
| 28 | NSSNKPAVTTKSPAVKPAAAPKQPVGGGQKLLTRKADSSSSEEESSSSEEEKTKKMVATTKPKATAKAALSLPAKQAPQGSRDSSSDSDSSSSEEEEEKTSKSAVKKKPQKVAGGAAPSKPASAKKGKAESSNSSSSDDSSEEEEEKLKGKGSPRPQAPKANGTSALTAQNGKAAKNSEEEEEEKKKAAVVVSKSGSLKKRKQNEAAKEAETPQAKKIKLAAALE- | Homo sapiens nucleolar and coiled-body phosphoprotein 1 (NOLC1) |
| 29 | NSSNKPAVTTKSPAVKPAAAPKQPVGGGQKLLTRKADSSSSEEESSSSEEEKTKKMVATTKPKATAKAALSLPAKQAPQGSRDSSSDSDSSSSEEEEEKTSKSAVKKKPQKVAGGAAPSKPASAKKGKAESSNSSSSDDSSEEEEEKLKGKGSPRPQAPKANGTSALTAQNGKAAKNSEEEEEEKKKAAVVVSKSGSLKKRKQNEAAKEAETPQAKKIKLAAALE- | Homo sapiens nucleolar and coiled-body phosphoprotein 1 (NOLC1) |
| 30 | NSSNKPAVTTKSPAVKPAAAPKQPVGGGQKLLTRKADSSSSEEESSSSEEEKTKKMVATTKPKATAKAALSLPAKQAPQGSRDSSSDSDSSSSEEEEEKTSKSAVKKKPQKVAGGAAPSKPASAKKGKAESSNSSSSDDSSEEEEEKLKGKGSPRPQAPKANGTSALTAQNGKAAKNSEEEEEEKKKAAVVVSKSGSLKKRKQNEAAKEAETPQAKKIKLAAALE- | Homo sapiens nucleolar and coiled-body phosphoprotein 1 (NOLC1) |
| 31 | NSSNKPAVTTKSPAVKPAAAPKQPVGGGQKLLTRKADSSSSEEESSSSEEEKTKKMVATTKPKATAKAALSLPAKQAPQGSRDSSSDSDSSSSEEEEEKTSKSAVKKKPQKVAGGAAPSKPASAKKGKAESSNSSSSDDSSEEEEEKLKGKGSPRPQAPKANGTSALTAQNGKAAKNSEEEEEEKKKAAVVVSKSGSLKKRKQNEAAKEAETPQAKKIKLAAALE- | Homo sapiens nucleolar and coiled-body phosphoprotein 1 (NOLC1) |
| 32 | NSSGKGEKEPPSPGMERKRSRRRGVGADPEARAEAGEQPGTAERALLRDQPRGRGQRGARQRRRTPRPLTSARAKAANVQEPEKKKKRRE- | Homo sapiens ribosomal RNA processing 1 homolog (S. cerevisiae)  (RRP1) |
| 33 | NSSKKRRQRRRGGGGRGRRKRGAAAAL- | genome sequence |
| 34 | NSSNKPAVTTKSPAVKPAAAPKQPVGGGQKLLTRKADSSSSEEESSSSEEEKTKKMVATTKPKATAKAALSLPAKQAPQGSRDSSSDSDSSSSEEEEEKTSKSAVKKKPQKVAGGAAPSKPASAKKGKAESSNSSSSDDSSEEEEEKLKGKGSPRPQAPKANGTSALTAQNGKAAKNSEEEEEEKKKAAVVVSKSGSLKKRKQNEAAKEAETPQAKKIKLAAALE- | Homo sapiens nucleolar and coiled-body phosphoprotein 1 (NOLC1) |
| 35 | NSSDESNKETANLQERSISNDDGEEKNSNKCASERKKTQTFSHCIR- | frameshift |
| 36 | NSSRRPSPHDEEEFEVEEAAEAQAEPKDGSPEAPASPEREEVALSEYKTETYDDYKDETTIDDSIMDADSLWVDTQDDDRSIMTEQLETIPKEEKAEKEARRSSLEKHRKEKPFKTGRGRISTPERKVAKKEPSTVSRDEVRRKKLAAALE- | Homo sapiens microtubule-associated protein 2 (MAP2), |
| 37 | NSSNKPAVTTKSPAVKPAAAPKQPVGGGQKLLTRKADSSSSEEESSSSEEEKTKKMVATTKPKATAKAALSLPAKQAPQGSRDSSSDSDSSSSEEEEEKTSKSAVKKKPQKVAGGAAPSKPASAKKGKAESSNSSSSDDSSEEEEEKLKGKGSPRPQAPKANGTSALTAQNGKAAKNSEEEEEEKKKAAVVVSKSGSLKKRKQNEAAKEAETPQAKKIKLAAALE- | Homo sapiens nucleolar and coiled-body phosphoprotein 1 (NOLC1) |
| 38 | NSQEDSEDSEDKDVKTKKDDSHSAEDSEDEKEDHKNVRQQRQAASKAASKQREMLMEDVGSEEEQEEEDEAPFQEKDSGSDEDFLMEDDDDSDYGSSKKKNKKMVKKSKPERKEKKMPKPRLKATVTPSPVKGKGKVGRPTASKASKEKTPSPKEEDEEPESLRPHSSN- | Homo sapiens nuclear casein kinase and cyclin-dependent kinase substrate 1 (NUCKS1) |
| 39 | NSQEDSEDSEDKDVKTKKDDSHSAEDSEDEKEDHKNVRQQRQAASKAASKQREMLMEDVGSEEEQEEEDEAPFQEKDSGSDEDFLMEDDDDSDYGSSKKKNKKMVKKSKPERKEKKMPKPRLKATVTPSPVKGKGKVGRPTASKASKEKTPSPKEEDEEPEACGRTRVTS- | Homo sapiens nuclear casein kinase and cyclin-dependent kinase substrate 1 (NUCKS1) |
| 40 | NSRTNFPITRTTEAYNPIIP- | reversed cDNA sequence |
| 41 | NSLMLMLNFMEK- | non-coding region of the cDNA |
| 42 | NSSVFY- | genome sequence |
| 43 | NSSNKPAVTTKSPAVKPAAAPKQPVGGGQKLLTRKADSSSSEEESSSSEEEKTKKMVATTKPKATAKAALSLPAKQAPQGSRDSSSDSDSSSSEEEEEKTSKSAVKKKPQKVAGGAAPSKPASAKKGKAESSNSSSSDDSSEEEEEKLKGKGSPRPQAPKANGTSALTAQNGKAAKNSEEEEEEKKKAAVVVSKSGSLKKRKQNEAAKEAETPQAKKIKLAAALE- | Homo sapiens nucleolar and coiled-body phosphoprotein 1 (NOLC1) |

: initial parts of the sequence that originate from the T7 phage.
