# Supplementary material for: Screening E3 Substrates Using a Live Phage Display Library
Source: PLoS One. 2013 Oct 4;8(10):e76622. doi: 10.1371/journal.pone.0076622 (PMC3790729; doi:10.1371/journal.pone.0076622)
Supplement: Table S2 — Encoding sequence and encoding protein of clones selected in Experiment 2. (DOC) [file pone.0076622.s003.doc]

Table S2: Encoding sequence and encoding protein of clones selected in Experiment 2

| Serial number | Encoding peptide sequence | Encoding protein |
| --- | --- | --- |
| 1 | NSSNKPAVTTKSPAVKPAAAPKQPVGGGQKLLTRKADSSSSEEESSSSEEEKTKKMVATTKPKATAKAALSLPAKQAPQGSRDSSSDSDSSSSEEEEEKTSKSAVKKKPQKVAGGAAPSKPASAKKGKAESSNSSSSDDSSEEEEEKLKGKGSPRPQAPKANGTSALTAQNGKAAKNSEEEEEEKKKAAVVVSKSGSLKKRKQNEAAKEAETPQAKKIKLAAALE- | Homo sapiens nucleolar and coiled-body phosphoprotein 1 (NOLC1), |
| 2 | NSISNVNQWGEPQLQERFLS- | non-coding region of the cDNA |
| 3 | NSSNYWNGLFKWILKYCYV- | non-coding region of the cDNA |
| 4 | NSS- | frameshift |
| 5 | NSSNKPAVTTKSPAVKPAAAPKQPVGGGQKLLTRKADSSSSEEESSSSEEEKTKKMVATTKPKATAKAALSLPAKQAPQGSRDSSSDSDSSSSEEEEEKTSKSAVKKKPQKVAGGAAPSKPASAKKGKAESSNSSSSDDSSEEEEEKLKGKGSPRPQAPKANGTSALTAQNGKAAKNSEEEEEEKKKAAVVVSKSGSLKKRKQNEAAKEAETPQAKKIKLAAALE- | Homo sapiens nucleolar and coiled-body phosphoprotein 1 (NOLC1), |
| 6 | NSTKEITPFILIPAKKKKKKKGGGKFLIFFPKPRFFPPPNPGF- | genome sequence |
| 7 | NSS- | genome sequence |
| 8 | NSSPKPGRIRI- | genome sequence |
| 9 | NSRPAWTTKQDPLQKQKKHTHTQKMSWVWWHTPVVRATWEAEAEGLLEPRS- | genome sequence |
| 10 | NSRETEEKTQETIKKQ- | genome sequence |
| 11 | NSSLHPFTLPLWLLCVIGLLKREDIWFLFFFFFFPAGPLALLKGASISFTFKINIDRGGFEPARILLPCTFVSPIAWFL- | genome sequence |
| 13 | NSSVNPGGGACSEPRSPHCTPAWATE- | genome sequence |
| 15 | NSSEIFGGGDQ- | non-coding region of the cDNA |
| 16 | NSRPYVRSTGTETGSNINVNSELNPSTGNRSREQSSEAAETGVSENEENPVRIISVTPVKNIDPVKNKEINSDQATQGNISSDRGKKRTVTAAGAENIQQKTDEKVDESGPPAPSKPRRGRRPKSESQGNATKNDDLNKPINKGRKRAAVGQESPGGLEAGNAKAPKLQDLAKKAAPAERQIDLQR- | Homo sapiens PDS5, regulator of cohesion maintenance, homolog |
| 17 | NSSARHRLKKKKKKKWH- | no match |
| 18 | NSSNKPAVTTKSPAVKPAAAPKQPVGGGQKLLTRKADSSSSEEESSSSEEEKTKKMVATTKPKATAKAALSLPAKQAPQGSRDSSSDSDSSSSEEEEEKTSKSAVKKKPQKVAGGAAPSKPASAKKGKAESSNSSSSDDSSEEEEEKLKGKGSPRPQAPKANGTSALTAQNGKAAKNSEEEEEEKKKAAVVVSKSGSLKKRKQNEAAKEAETPQAKKIKLAAALE- | Homo sapiens nucleolar and coiled-body phosphoprotein 1 (NOLC1), |
| 19 | NSF- | reversed cDNA sequence |
| 20 | NSSLICNLLPTKLHSARRGGSCLSSQGHFPTSSSASARTTEPDRQPYLYLKTKQNKTIS- | genome sequence |
| 21 | NSSSATQVAGIA- | non-coding region of the cDNA |
| 22 | NSSAEQAPPQGNVGPPFTL- | no match |
| 23 | NSPFTRGRREDYVGGQSHRSRNIGSNYPEKLSARDGHNQKDNTKSKEKESENAPGDGKGNKHKKHRKRRKGEESEGFLNPELLETSRKSREPTGVEENKTDSLFVLPSRDDATPVRDEPMDAESITFKSVSEKDKRERDKPKAKGDKTKRKNDGSAVSKKENIVKPAKGPQEKVDGERERSPRSEACGRTRVTS- | Homo sapiens retinoblastoma binding protein 6 (RBBP6), |
| 24 | NSSKSFSK- | reversed cDNA sequence |
| 25 | NSSISNSYNTTNQVIVFMTKELRQVIAWFPFVKLLQY- | reversed cDNA sequence |
| 26 | NSSCFNLNKIVVFPAPSNPNVTTRISIFGPM- | reversed cDNA sequence |
| 27 | NSFFILDLGTLIINALK- | non-coding region of the cDNA |
| 29 | NSSLEFKLSSRNAVVVRII- | reversed cDNA sequence |
| 30 | NSSIIRRMSWSPRHSSKPPYSSRAGPARQPSR- | genome sequence |
| 31 | NSSKKSKNKQKKKKLYLNT- | genome sequence |
| 32 | NSSGIWGTWSSIACPKAKATKSVQKK- | non-coding region of the cDNA |
| 33 | NSSGEWHEPRRQSLQ- | no match |
| 34 | NSIKHTKMT- | genome sequence |
| 35 | NSSLKRKKDGRKRRERMGTTRGRKKGSKV- | genome sequence |
| 36 | NSSQIKHNSRSI- | reversed cDNA sequence |
| 37 | NSY- | non-coding region of the cDNA |
| 39 | NSSSQRKRTRRRTKLRSL- | frameshifts |
| 40 | NSSKNIKQKKKINLTLSKFKTSIHQKT- | reversed cDNA sequence |
| 41 | NSS- | genome sequence |
| 42 | NSSLHWWDQG- | genome sequence |
| 43 | NSSNKPAVTTKSPAVKPAAAPKQPVGGGQKLLTRKADSSSSEEESSSSEEEKTKKMVATTKPKATAKAALSLPAKQAPQGSRDSSSDSDSSSSEEEEEKTSKSAVKKKPQKVAGGAAPSKPASAKKGKAESSNSSSSDDSSEEEEEKLKGKGSPRPQAPKANGTSALTAQNGKAAKNSEEEEEEKKKAAVVVSKSGSLKKRKQNEAAKEAETPQAKKIKLAAALE- | Homo sapiens nucleolar and coiled-body phosphoprotein 1 (NOLC1), |
| 44 | NSGS- | reversed cDNA sequence |

: initial parts of the sequence that originate from the T7 phage.
