# Supplementary material for: Screening E3 Substrates Using a Live Phage Display Library
Source: PLoS One. 2013 Oct 4;8(10):e76622. doi: 10.1371/journal.pone.0076622 (PMC3790729; doi:10.1371/journal.pone.0076622)
Supplement: Table S3 — Encoding sequence and encoding protein of clones selected in Experiment 3. (DOC) [file pone.0076622.s004.doc]

Table S3: Encoding sequence and encoding protein of clones selected in Experiment 3

| Serial number | Encoding peptide sequence | Encoding protein |
| --- | --- | --- |
| 1-1 | NSPQPPARRGKKS- | genome sequence |
| 1-2 | NSSRSMS- | non-coding region of the cDNA |
| 1-3 | NSVPTNSAQQGHNSPDSPVTSAAKGIPGFGNTGNISGAPVTYPSAGAQGVNNTASGNNSREGTGGSNGKRERYTENRGSSRHSHGETGNRHSDSPRHGDGGRHGDGYRHPESSSRHTDGHRHGENRHGGSAGRHGENRGANDGRNGESRKEACGRTRVTS- | Homo sapiens DEAD (Asp-Glu-Ala-Asp) box polypeptide 42 (DDX42) |
| 1-4 | NSSRRYDRKQSGYGGQTKPIFRKKAKTTKKIVLRLECVEPNCRSKRMLAIKRCKHFELGGDKKRKGQVIQF- | Homo sapiens ribosomal protein L36a (RPL36A) |
| 1-5 | NSVPTNSAQQGHNSPDSPVTSAAKGIPGFGNTGNISGAPVTYPSAGAQGVNNTASGNNSREGTGGSNGKRERYTENRGSSRHSHGETGNRHSDSPRHGDGGRHGDGYRHPESSSRHTDGHRHGENRHGGSAGRHGENRGANDGRNGESRKEACGRTRVTS- | Homo sapiens DEAD (Asp-Glu-Ala-Asp) box polypeptide 42 (DDX42) |
| 1-6 | NSSNKPAVTTKSPAVKPAAAPKQPVGGGQKLLTRKADSSSSEEESSSSEEEKTKKMVATTKPKATAKAALSLPAKQAPQGSRDSSSDSDSSSSEEEEEKTSKSAVKKKPQKVAGGAAPSKPASAKKGKAESSNSSSSDDSSEEEEEKLKGKGSPRPQAPKANGTSALTAQNGKAAKNSEEEEEEKKKAAVVVSKSGSLKKRKQNEAAKEAETPQAKKIKLAAALE- | Homo sapiens nucleolar and coiled-body phosphoprotein 1 (NOLC1), |
| 1-7 | NSSRGHGRDGVHRGREQHERPGVRVPAVPGRHGRRTRGVRGGGGRGRGLKTSQINRASLVNFCCPQAWSFYL- | frameshift |
| 1-9 | NSSRGHGRDGVHRGREQHERPGVRVPAVPGRHGRRTRGVRGGGGRGRGLKTSQINRASLVNFCCPQAWSFYL- | frameshift |
| 1-10 | NSVQLTQHNRAITVLTAPSPVPPRASQALAILATSVVPL- | frameshift |
| 1-11 | NSVQLTQHNRAITVLTAPSPVPPRASQALAILATSVVPL- | frameshift |
| 1-12 | NSVQLTQHNRAITVLTAPSPVPPRASQALAILATSVVPL- | frameshift |
| 1-13 | NSSLYVLEKAFLSTHPNTETVFEAFLKSYSTSSKKARPVLKKLDEVRLRGRKRSMVG- | TP53RK |
| 1-15 | NSSRGHGRDGVHRGREQHERPGVRVPAVPGRHGRRTRGVRGGGGRGRGLKTSQINRASLVNFCCPQAWSFYL- | frameshift |
| 1-16 | NSSGRKEKRERTEKEEERKREREGGRKEGKEKEEKEEKKDRKERKKRKGRKERKEGGKENKTWQKKRQCKY- | genome sequence |
| 1-17 | NSFLHAQDVLLNETLSACFKWIF- | non-coding region of the cDNA |
| 1-18 | NSSGGESQEGAPEIPPGGGRQGMRCREARALPSGGREKSAISPGGSRRKGLALAALKSLKKWHGMTQKVPFWDHKHPERYFP- | genome sequence |
| 1-19 | NSSNKPAVTTKSPAVKPAAAPKQPVGGGQKLLTRKADSSSSEEESSSSEEEKTKKMVATTKPKATAKAALSLPAKQAPQGSRDSSSDSDSSSSEEEEEKTSKSAVKKKPQKVAGGAAPSKPASAKKGKAESSNSSSSDDSSEEEEEKLKGKGSPRPQALKANGTSALTAQNGKAAKNSEEEEEEKKKAAVVVSKSGSLKKRKQNEAAKEAETPQAKKIKLAAALE- | Homo sapiens nucleolar and coiled-body phosphoprotein 1 (NOLC1), |
| 1-23 | NSVPTNSAQQGHNSPDSPVTSAAKGIPGFGNTGNISGAPVTYPSAGAQGVNNTASGNNSREGTGGSNGKRERYTENRGSSRHSHGETGNRHSDSPRHGDGGRHGDGYRHPESSSRHTDGHRHGENRHGGSAGRHGENRGANDGRNGESRKEACGRTRVTS- | Homo sapiens DEAD (Asp-Glu-Ala-Asp) box polypeptide 42 (DDX42) |
| 1-24 | NSVPTNSAQQGHNSPDSPVTSAAKGIPGFGNTGNISGAPVTYPSAGAQGVNNTASGNNSREGTGGSNGKRERYTENRGSSRHSHGETGNRHSDSPRHGDGGRHGDGYRHPESSSRHTDGHRHGENRHGGSAGRHGENRGANDGRNGESRKEACGRTRVTS- | Homo sapiens DEAD (Asp-Glu-Ala-Asp) box polypeptide 42 (DDX42) |
| 1-25 | NSYWVGEDSTYKFFEVILIDPFHKAIRRNPDTQWITKPVHKHREMRGLTSAGRKSRGLGKGHKFHHTIGGSRRAAWRRRNTLQLHRYR- | Homo sapiens ribosomal protein L15 (RPL15), |
| 1-26 | NSSGYLHFPWMEHRRKEKTRDRPKKKKIRCKRNE- | genome sequence |
| 1-27 | NSHNSV- | non-coding region of the cDNA |
| 1-28 | NSSKITRTRESEVAVSQDSAISLQPGDRVRLRFKKKKKKAGGPPQLTI- | no match |
| 1-29 | NSTLQARRSLKMAVSS--- | non-coding region of the cDNA |
| 1-30 | NSVPTNSAQQGHNSPDSPVTSAAKGIPGFGNTGNISGAPVTYPSAGAQGVNNTASGNNSREGTGGSNGKRERYTENRGSSRHSHGETGNRHSDSPRHGDGGRHGDGYRHPESSSRHTDGHRHGENRHGGSAGRHGENRGANDGRNGESRKEACGRTRVTS- | Homo sapiens DEAD (Asp-Glu-Ala-Asp) box polypeptide 42 (DDX42) |
| 1-31 | NSVPTNSAQQGHNSPDSPVTSAAKGIPGFGNTGNISGAPVTYPSAGAQGVNNTASGNNSREGTGGSNGKRERYTENRGSSRHSHGETGNRHSDSPRHGDGGRHGDGYRHPESSSRHTDGHRHGENRHGGSAGRHGENRGANDGRNGESRKEACGRTRVTS- | Homo sapiens DEAD (Asp-Glu-Ala-Asp) box polypeptide 42 (DDX42) |
| 1-32 | NSVPTNSAQQGHNSPDSPVTSAAKGIPGFGNTGNISGAPVTYPSAGAQGVNNTASGNNSREGTGGSNGKRERYTENRGSSRHSHGETGNRHSDSPRHGDGGRHGDGYRHPESSSRHTDGHRHGENRHGGSAGRHGENRGANDGRNGESRKEACGRTRVTS- | Homo sapiens DEAD (Asp-Glu-Ala-Asp) box polypeptide 42 (DDX42) |
| 1-33 | NSVPTNSAQQGHNSPDSPVTSAAKGIPGFGNTGNISGAPVTYPSAGAQGVNNTASGNNSREGTGGSNGKRERYTENRGSSRHSHGETGNRHSDSPRHGDGGRHGDGYRHPESSSRHTDGHRHGENRHGGSAGRHGENRGANDGRNGESRKEACGRTRVTS- | Homo sapiens DEAD (Asp-Glu-Ala-Asp) box polypeptide 42 (DDX42) |
| 1-34 | NSSGWLTSWAGRRKHTHIHTH- | genome sequence |
| 1-35 | NSSKYCIILSKDKKKKKKKKKKKKKKKKKKKKKKKKFFLAPPPRFFFGGNPPKKRGKTIFPPPKKFWGPPQKKNTPPGGPKGGGGG- | non-coding region of the cDNA |
| 1-36 | NSYWVGEDSTYKFFEVILIDPFHKAIRRNPDTQWITKPVHKHREMRGLTSAGRKSRGLGKGHKFHHTIGGSRRAAWRRRNTLQLHRYR- | Homo sapiens ribosomal protein L15 (RPL15), |
| 1-37 | NSFHI- | non-coding region of the cDNA |
| 1-38 | NSSKGNTAFFFFKNPTHSPSPSYLDDLGSYLTKLNKKHLLGQAWRPMPVIPALFGRLKQADHEVRKLKPPWLTR- | genome sequence |
| 1-39 | NSSDP- | non-coding region of the cDNA |
| 1-40 | NSSGYLHFPWMEHRRKEKTRDRPKKKKIRCKRNE- | genome sequence |
| 1-41 | NSRPVWATWQDSISIQKKKKKERKKEKKTLSLFKKRKNEKN- | genome sequence |
| 1-42 | NSSEPPPEPKRRRVGDVEPSRKPKRRRAADVEPSSPEPKRRRVGDVEPSRKPKRRRAADVEPSSPEPKRRRVGDVEPSRKPNKRRAADVEPSLPEPKRRRLS- | no match |
| 1-43 | NSVPTNSAQQGHNSPDSPVTSAAKGIPGFGNTGNISGAPVTYPSAGAQGVNNTASGNNSREGTGGSNGKRERYTENRGSSRHSHGETGNRHSDSPRHGDGGRHGDGYRHPESSSRHTDGHRHGENRHGGSAGRHGENRGANDGRNGESRKEACGRTRVTS- | Homo sapiens DEAD (Asp-Glu-Ala-Asp) box polypeptide 42 (DDX42), |
| 1-44 | NSVPTNSAQQGHNSPDSPVTSAAKGIPGFGNTGNISGAPVTYPSAGAQGVNNTASGNNSREGTGGSNGKRERYTENRGSSRHSHGETGNRHSDSPRHGDGGRHGDGYRHPESSSRHTDGHRHGENRHGGSAGRHGENRGANDGRNGESRKEACGRTRVTS- | Homo sapiens DEAD (Asp-Glu-Ala-Asp) box polypeptide 42 (DDX42), |
| 2-1 | NSYWVGEDSTYKFFEVILIDPFHKAIRRNPDTQWITKPVHKHREMRGLTSAGRKSRGLGKGHKFHHTIGGSRRAAWRRRNTLQLHRYR- | Homo sapiens ribosomal protein L15 (RPL15) |
| 2-2 | NSIILRKRKLCGPIMLNA- | reversed cDNA sequences |
| 2-3 | NSSNKPAVTTKSPAVKPAAAPKQPVGGGQKLLTRKADSSSSEEESSSSEEEKTKKMVATTKPKATAKAALSLPAKQAPQGSRDSSSDSDSSSSEEEEEKTSKSAVKKKPQKVAGGAAPSKPASAKKGKAESSNSSSSDDSSEEEEEKLKGKGSPRPQAPKANGTSALTAQNGKAAKNSEEEEEEKKKAAVVVSKSGSLKKRKQNEAAKEAETPQAKKIKLAAALE- | Homo sapiens nucleolar and coiled-body phosphoprotein 1 (NOLC1), |
| 2-4 | NSVPTNSAQQGHNSPDSPVTSAAKGIPGFGNTGNISGAPVTYPSAGAQGVNNTASGNNSREGTGGSNGKRERYTENRGSSRHSHGETGNRHSDSPRHGDGGRHGDGYRHPESSSRHTDGHRHGENRHGGSAGRHGENRGANDGRNGESRKEACGRTRVTS- | Homo sapiens DEAD (Asp-Glu-Ala-Asp) box polypeptide 42 (DDX42), |
| 2-5 | NSVPTNSAQQGHNSPDSPVTSAAKGIPGFGNTGNISGAPVTYPSAGAQGVNNTASGNNSREGTGGSNGKRERYTENRGSSRHSHGETGNRHSDSPRHGDGGRHGDGYRHPESSSRHTDGHRHGENRHGGSAGRHGENRGANDGRNGESRKEACGRTRVTS- | Homo sapiens DEAD (Asp-Glu-Ala-Asp) box polypeptide 42 (DDX42), |
| 2-6 | NSSGI- | frameshift |
| 2-7 | NSSGKKKKAN- | reversed cDNA sequence |
| 2-8 | NSSQGRTLKISINSQLLFSLKVFLYLKLLSVLLFHCFKDFYNGCYLIAVFVEFATVIKYLWSTHSFAVRSYFKMFFGIYRNRF- | genome sequence |
| 2-9 | NSSGRPPRERRVDGIKGVSHVSLFQVRDSSRVVVVDACNPSYLGG- | genome sequence |
| 2-10 | NSSAYHKKCDQKKKDYEVELLRFLESLPEEEQQRVLGEEKMLNINKKQATSPASKKPAQEGGKGGSEKPKRPVSAMFIFSEEKRRQLQEERPELSESELTRLLARMWNDLSEKKKAKYKAREAALKAQSERKPGGEREERGKLPESPKRAEEIWQQSVIGDYLARFKNDRVKALKAMEMTWNNMEKKEKLMWIKKAAEDQKRYERELSEMRAPPAATNSSKKMKFQGEPKKPPMNGYQKFSQELLSNGELNHLPLKERMVEIGSRWQRISQSQKEHYKKLAEEQQKQYKVHLDLWVKSLSPQDRAAYKEYISNKRKSMTKLRGPNPKSSRDYSAVQVGVRGG- | Homo sapiens upstream binding transcription factor, RNA polymerase  I (UBTF) |
| 2-11 | NSSAYHKKCDQKKKDYEVELLRFLESLPEEEQQRVLGEEKMLNINKKQATSPASKKPAQEGGKGGSEKPKGPVSAMFIFSEEKRRQLQEERSELSQSELSRLQARLWNDLSETKKANDEVREAAFTDQLDVKLGGELSDLSKPPEV- | Homo sapiens upstream binding transcription factor, RNA polymerase  I (UBTF) |
| 2-12 | NSSKITRTRESEVAVSQDSAISLQPGDRVRLRFKKKKKKACAPPRLTS- | no match |
| 2-13 | NS- | genome sequence |
| 2-14 | NSSTGMSHHARQEIQLLKPQTQIRSTVGRSNGYGRNHLRRRCCSSCHQDGRQHC- | reversed cDNA sequence |
| 2-15 | NSHCPASVHLANAYFASQSQRIGKGS- | non-coding RNA |
| 2-16 | NSSEGQGEATVLEHLCQAAQSLQENRCLQLWLK- | genome sequence |
| 2-17 | NSSDRRKWPNRKQLLKPRNRKWKAQNRLRLSISLLET- | frameshift |
| 2-18 | NSSRRRRQSLWLRPRLRNHCSPPRPEPEHPGSRQPLTLPAKWARRRPTSTSWSSATWTPESPPPRATSSTNAEVLTKGPLRSSRRRRLRWGRDPSSMPGCWTS- | frameshift |
| 2-19 | NSSNKPAVTTKSPAVKPAAAPKQPVGGGQKLLTRKADSSSSEEESSSSEEEKTKKMVATTKPKATAKAALSLPAKQAPQGSRDSSSDSDSSSSEEEEEKTSKSAVKKKPQKVAGGAAPSKPASAKKGKAESSNSSSSDDSSEEEEEKLKGKGSPRPQAPKANGTSALTAQNGKAAKNSEEEEEEKKKAAVVVSKSGSLKKRKQNEAAKEAETPQAKKIKLAAALE- | Homo sapiens nucleolar and coiled-body phosphoprotein 1 (NOLC1), |
| 2-20 | NSSW- | genome sequence |
| 2-21 | NSAPPQAADFLCYACR- | frameshift |
| 2-22 | NSSEVTVLRSLLCPAWRASVKSSTPSTSHLEAGRSRR- | frameshift |
| 2-23 | NSSGGESQEGAPEIPPGGGRQGMRCREARALPSGGREKSAISPGGSRRKGLALAALKSLKKWHGMTQKVPFWDHKHPERYFP- | genome sequence |
| 2-24 | NSVPTNSAQQGHNSPDSPVTSAAKGIPGFGNTGNISGAPVTYPSAGAQGVNNTASGNNSREGTGGSNGKRERYTENRGSSRHSHGETGNRHSDSPRHGDGGRHGDGYRHPESSSRHTDGHRHGENRHGGSAGRHGENRGANDGRNGESRKEACGRTRVTS- | Homo sapiens DEAD (Asp-Glu-Ala-Asp) box polypeptide 42 (DDX42), |
| 2-25 | NSSKR- | frameshift |
| 2-26 | NSSGKKEKSKLKIQMTRDKATTGKRTHQKLG- | reversed cDNA sequence |
| 2-27 | NSSDVGSLQPWPSGLKQSSCLSLPNR- | genome sequence |
| 2-28 | NSNLCQPENTRGGWLEA- | genome sequence |
| 2-29 | NSVPTNSAQQGHNSPDSPVTSAAKGIPGFGNTGNISGAPVTYPSAGAQGVNNTASGNNSREGTGGSNGKRERYTENRGSSRHSHGETGNRHSDSPRHGDGGRHGDGYRHPESSSRHTDGHRHGENRHGGSAGRHGENRGANDGRNGESRKEACGRTRVTS- | Homo sapiens DEAD (Asp-Glu-Ala-Asp) box polypeptide 42 (DDX42), |
| 2-30 | NSVPTNSAQQGHNSPDSPVTSAAKGIPGFGNTGNISGAPVTYPSAGAQGVNNTASGNNSREGTGGSNGKRERYTENRGSSRHSHGETGNRHSDSPRHGDGGRHGDGYRHPESSSRHTDGHRHGENRHGGSAGRHGENRGANDGRNGESRKEACGRTRVTS- | Homo sapiens DEAD (Asp-Glu-Ala-Asp) box polypeptide 42 (DDX42), |
| 2-31 | NSVPTNSAQQGHNSPDSPVTSAAKGIPGFGNTGNISGAPVTYPSAGAQGVNNTASGNNSREGTGGSNGKRERYTENRGSSRHSHGETGNRHSDSPRHGDGGRHGDGYRHPESSSRHTDGHRHGENRHGGSAGRHGENRGANDGRNGESRKEACGRTRVTS- | Homo sapiens DEAD (Asp-Glu-Ala-Asp) box polypeptide 42 (DDX42), |
| 2-32 | NSVPTNSAQQGHNSPDSPVTSAAKGIPGFGNTGNISGAPVTYPSAGAQGVNNTASGNNSREGTGGSNGKRERYTENRGSSRHSHGETGNRHSDSPRHGDGGRHGDGYRHPESSSRHTDGHRHGENRHGGSAGRHGENRGANDGRNGESRKEACGRTRVTS- | Homo sapiens DEAD (Asp-Glu-Ala-Asp) box polypeptide 42 (DDX42), |
| 2-33 | NSHFYSLIKKLLPWPGAVAHACYPSTLGGQGGRITRSGDPDHPG- | genome sequence |
| 2-34 | NSVPTNSAQQGHNSPDSPVTSAAKGIPGFGNTGNISGAPVTYPSAGAQGVNNTASGNNSREGTGGSNGKRERYTENRGSSRHSHGETGNRHSDSPRHGDGGRHGDGYRHPESSSRHTDGHRHGENRHGGSAGRHGENRGANDGRNGESRKEACGRTRVTS- | Homo sapiens DEAD (Asp-Glu-Ala-Asp) box polypeptide 42 (DDX42), |
| 2-35 | NSSRGHGRDGVHRGREQHERPGVRVPAVPGRHGRRTRGVRGGGGRGRGLKTSQINRASLVNFCCPQAWSFYL- | frameshift |
| 2-36 | NSVPTNSAQQGHNSPDSPVTSAAKGIPGFGNTGNISGAPVTYPSAGAQGVNNTASGNNSREGTGGSNGKRERYTENRGSSRHSHGETGNRHSDSPRHGDGGRHGDGYRHPESSSRHTDGHRHGENRHGGSAGRHGENRGANDGRNGESRKEACGRTRVTS- | Homo sapiens DEAD (Asp-Glu-Ala-Asp) box polypeptide 42 (DDX42), |
| 2-37 | NSSADDNIKTPAERLRGPLPPSADDNLKTPSERQLTPLPPSAPPSADDNIKTPAERLRGPLPPSADDNLKTPSERQLAPLPPSPPPSANDNIKTPAERLRGPLPPSADDNLKTPPLTTQEAEAEKPPKPKRQRAAEMEPPPEPKRRRVGDVEPSRKPKRRRAADVEPSSPEPKRRRVGDVEPSRKPKRRRAADVEPSSPEPKRRRVGDVEPSRKPKRRRAADVEPSLPEPKRRRLS- | Homo sapiens nuclear pore complex-interacting protein-like  3-like, transcript variant 1 （NPIPL3） |
| 2-38 | NSSKKKKKKKKKKKKKKKKKKKKKKKKKKKKKKKKKKKKKKKRGGGPPPVFFPPPGGGGGGGGGGV- | no match |
| 2-39 | NSYWVGEDSTYKFFEVILIDPFHKAIRRNPDTQWITKPVHKHREMRGLTSAGRKSRGLGKGHKFHHTIGGSRRAAWRRRNTLQLHRYR- | Homo sapiens ribosomal protein L15 (RPL15), |
| 2-40 | NSSEELASQCVKLIDSASSHTSKLRRYREVRGSLWGNPPGTSKPGHV- | reversed cDNA sequence |
| 2-41 | NSSNKPAVTTKSPAVKPAAAPKQPVGGGQKLLTRKADSSSSEEESSSSEEEKTKKMVATTKPKATAKAALSLPAKQAPQGSRDSSSDSDSSSSEEEEEKTSKSAVKKKPQKVAGGAAPSKPASAKKGKAESSNSSSSDDSSEEEEEKLKGKGSPRPQAPKANGTSALTAQNGKAAKNSEEEEEEKKKAAVVVSKSGSLKKRKQNEAAKEAETPQAKKIKLAAALE- | Homo sapiens nucleolar and coiled-body phosphoprotein 1 (NOLC1), |
| 2-42 | NSSLLRGVDPQQREDGRVRHPAPRPEDVGHLHRQQHGHPGAVQAHLRAVHGHVPAQGLPALVHGRGHGRDGVHRGREQHERPGVRVPAVPGRHGRRTRGVRGGGGRGRGLKTSQINRASLVNFCCPQAWSFYL- | frameshift |
| 2-43 | NSSRQTRLLKKLKK- | frameshift |
| 2-44 | NSSQEKKKKKKKKKKKKKNPPEGQPRKNFGF- | no match |
| 2-45 | NSSGYLHFPWMEHRRKEKTRDRPKKKKLRCKINE- | genome sequence |
| 2-46 | NSMFDSQ- | genome sequence |
| 2-47 | NSSRGHGRDGVHRGREQHERPGVRVPAVPGRHGRRTRGVRGGGGRGRGLKTSQINRASLVNFCCPQAWSFYL- | frameshift |
| 2-48 | NSSLLRGVDPQQREDGRVRHPAPRPEDVGHLHRQQHGHPGAVQAHLRAVHGHVPAQGLPALVHGRGHGRDGVHRGREQHERPGVRVPAVPGRHGRRTRGVRGGGGRGRGLKTSQINRASLVNFCCPQAWSFYL- | frameshift |
| 2-49 | NSPSIWFPMDGTWRTKSYKRQTQVKTIQKCFKQKMSTNQMIIHTQIIKIVKTLQEMPHEISMRETGFPISGRK- | genome sequence |
| 2-50 | NSSGKK- | reversed cDNA sequence |
| 2-51 | NSSGKKEKSKLKIQMTRDKATTGKRTHQKLG- | reversed cDNA sequence |
| 2-52 | NSAVHVLCLVTQEGRQQGDPRAAVGPRTRGWPGGPGRGKPPVPHMAQTGQGRGARRREGLRRRGCETERTEKRGH- | reversed cDNA sequence |
| 2-53 | NSMFDSQ- | genome sequence |
| 2-54 | NSSHRTGVQILRKPHNRRQKVASPQESGDKEEPVPDPALFPAGALRPQDSVGQGVGLGGEEGFGEKRDQEGKTGERQQKNPEEKTRKEKRDSGPAIGKDKKNHHRRERSKGEGERIGKKLAAALE- | reversed cDNA sequence |
| 2-55 | NSSISQLSPPSHLQ- | frameshift |
| 2-56 | NSSISQLSPPSHLQ- | frameshift |
| 2-57 | NSSEGQGEATVLEHLCQAAQSLQENRCLQLWLK- | genome sequence |
| 2-58 | NSSISQLSPPSHLQ- | frameshift |

: initial parts of the sequence that originate from the T7 phage.
