# Supplementary material for: Screening E3 Substrates Using a Live Phage Display Library
Source: PLoS One. 2013 Oct 4;8(10):e76622. doi: 10.1371/journal.pone.0076622 (PMC3790729; doi:10.1371/journal.pone.0076622)
Supplement: Table S4 — Encoding sequence and encoding protein of clones selected in Experiment 4. (DOC) [file pone.0076622.s005.doc]

Table S4: Encoding sequence and encoding protein of clones selected in Experiment 4

| Serial number | Encoding peptide sequence | Encoding protein |
| --- | --- | --- |
| 1 | NSSNKPAVTTKSPAVKPAAAPKQPVGGGQKLLTRKADSSSSEEESSSSEEEKTKKMVATTKPKATAKAALSLPAKQAPQGSRDSSSDSDSSSSEEEEEKTSKSAVKKKPQKVAGGAAPSKPASAKKGKAESSNSSSSDDSSEEEEEKLKGKGSPRPQAPKANGTSALTAQNGKAAKNSEEEEEEKKKAAVVVSKSGSLKKRKQNEAAKEAETPQAKKIKLAAALE- | Homo sapiens nucleolar and coiled-body phosphoprotein 1 (NOLC1), |
| 2 | NSSNKPAVTTKSPAVKPAAAPKQPVGGGQKLLTRKADSSSSEEESSSSEEEKTKKMVATTKPKATAKAALSLPAKQAPQGSRDSSSDSDSSSSEEEEEKTSKSAVKKKPQKVAGGAAPSKPASAKKGKAESSNSSSSDDSSEEEEEKLKGKGSPRPQAPKANGTSALTAQNGKAAKNSEEEEEEKKKAAVVVSKSGSLKKRKQNEAAKEAETPQAKKIKLAAALE- | Homo sapiens nucleolar and coiled-body phosphoprotein 1 (NOLC1), |
| 7 | NSATKLSEK- | genome sequence |
| 8 | NSAERCFDLGAR- | frameshift |
| 9 | NSSNKPAVTTKSPAVKPAAAPKQPVGGGQKLLTRKADSSSSEEESSSSEEEKTKKMVATTKPKATAKAALSLPAKQAPQGSRDSSSDSDSSSSEEEEEKTSKSAVKKKPQKVAGGAAPSKPASAKKGKAESSNSSSSDDSSEEEEEKLKGKGSPRPQAPKANGTSALTAQNGKAAKNSEEEEEEKKKAAVVVSKSGSLKKRKQNEAAKEAETPQAKKIKLAAALE- | Homo sapiens nucleolar and coiled-body phosphoprotein 1 (NOLC1), |
| 10 | NSLDCGVESAERTLCTVRPCQKVHFTF- | frameshift |
| 11 | NSSNKPAVTTKSPAVKPAAAPKQPVGGGQKLLTRKADSSSSEEESSSSEEEKTKKMVATTKPKATAKAALSLPAKQAPQGSRDSSSDSDSSSSEEEEEKTSKSAVKKKPQKVAGGAAPSKPASAKKGKAESSNSSSSDDSSEEEEEKLKGKGSPRPQAPKANGTSALTAQNGKAAKNSEEEEEEKKKAAVVVSKSGSLKKRKQNEAAKEAETPQAKKIKLAAALE- | Homo sapiens nucleolar and coiled-body phosphoprotein 1 (NOLC1), |
| 12 | NSSMAWKKVQ- | frameshift |
| 13 | NSSPKRRSARLSAKPPAKVEAKPKKAAAKDKSSDKKVQTKGKRGAKGKQAEVANQETKEDLPAENGETKTEESPASDEAGEKEAKSD-- | Homo sapiens high mobility group nucleosome binding domain 1 (HMGN1), |
| 14 | NSSNKPAVTTKSPAVKPAAAPKQPVGGGQKLLTRKADSSSSEEESSSSEEEKTKKMVATTKPKATAKAALSLPAKQAPQGSRDSSSDSDSSSSEEEEEKTSKSAVKKKPQKVAGGAAPSKPASAKKGKAESSNSSSSDDSSEEEEEKLKGKGSPRPQAPKANGTSALTAQNGKAAKNSEEEEEEKKKAAVVVSKSGSLKKRKQNEAAKEAETPQAKKIKLAAALE- | Homo sapiens nucleolar and coiled-body phosphoprotein 1 (NOLC1), |
| 18 | NSWTSSMPNNEPHPSSLPFLPSQGLSSPEAQ- | frameshift |
| 19 | NSSNKPAVTTKSPAVKPAAAPKQPVGGGQKLLTRKADSSSSEEESSSSEEEKTKKMVATTKPKATAKAALSLPAKQAPQGSRDSSSDSDSSSSEEEEEKTSKSAVKKKPQKVAGGAAPSKPASAKKGKAESSNSSSSDDSSEEEEEKLKGKGSPRPQAPKANGTSALTAQNGKAAKNSEEEEEEKKKAAVVVSKSGSLKKRKQNEAAKEAETPQAKKIKLAAALE- | Homo sapiens nucleolar and coiled-body phosphoprotein 1 (NOLC1), |
| 20 | NSYWVGEDSTYKFFEVILIDPFHKAIRRNPDTQWITKPVHKHREMRGLTSAGRKSRGLGKGHKFHHTIGGSRRAAWRRRNTLQLHRYR- | Homo sapiens ribosomal protein L15 (RPL15), |
| 21 | NSEKASQDTINSSVVEENGEVKELHPCKYCKKVFGTHTNMRRHQRRVHERHLIPKGVRRKGGLEEPQPPAEQAQATQNVYVPSTEPEEEGEADDVYIMDISSNIAAALE- | Homo sapiens PR domain containing 2, with ZNF domain（PRDM2） |
| 22 | NSSNKPAVTTKSPAVKPAAAPKQPVGGGQKLLTRKADSSSSEEESSSSEEEKTKKMVATTKPKATAKAALSLPAKQAPQGSRDSSSDSDSSSSEEEEEKTSKSAVKKKPQKVAGGAAPSKPASAKKGKAESSNSSSSDDSSEEEEEKLKGKGSPRPQAPKANGTSALTAQNGKAAKNSEEEEEEKKKAAVVVSKSGSLKKRKQNEAAKEAETPQAKKIKLAAALE- | Homo sapiens nucleolar and coiled-body phosphoprotein 1 (NOLC1), |
| 23 | NSMM- | frameshift |
| 24 | NSSNKPAVTTKSPAVKPAAAPKQPVGGGQKLLTRKADSSSSEEESSSSEEEKTKKMVATTKPKATAKAALSLPAKQAPQGSRDSSSDSDSSSSEEEEEKTSKSAVKKKPQKVAGGAAPSKPASAKKGKAESSNSSSSDDSSEEEEEKLKGKGSPRPQAPKANGTSALTAQNGKAAKNSEEEEEEKKKAAVVVSKSGSLKKRKQNEAAKEAETPQAKKIKLAAALE- | Homo sapiens nucleolar and coiled-body phosphoprotein 1 (NOLC1) |
| 25 | NSEVLGLSQKTVMYLKFLKYCKPTEFRLVLPKRKAT- | frameshift |
| 26 | NSSWPCNIV- | no match |
| 27 | NSSNKPAVTTKSPAVKPAAAPKQPVGGGQKLLTRKADSSSSEEESSSSEEEKTKKMVATTKPKATAKAALSLPAKQAPQGSRDSSSDSDSSSSEEEEEKTSKSAVKKKPQKVAGGAAPSKPASAKKGKAESSNSSSSDDSSEEEEEKLKGKGSPRPQAPKANGTSALTAQNGKAAKNSEEEEEEKKKAAVVVSKSGSLKKRKQNEAAKEAETPQAKKIKLAAALE- | Homo sapiens nucleolar and coiled-body phosphoprotein 1 (NOLC1), |
| 28 | NSSESISLTKLESSPRKLHKDKRQENKHKTFLPVKGNTEKSNMLEFKLCPDILLKNTNSVEERKDVKPHPRKEQAPLQVSGIKSTKEDWLKFVATKKRTQKDSQERDNVNSRLSKRSFSADGFEMLQNPVKDSKEMFQTYKQMYLEKRSRSLGSSPVK- | Homo sapiens chromosome 12 open reading frame 35 (C12orf35), |
| 29 | NSSESISLTKLESSPRKLHKDKRQENKHKTFLPVKGNTEKSNMLEFKLCPDILLKNTNSVEERKDVKPHPRKEQAPLQVSGIKSTKEDWLKFVATKKRTQKDSQERDNVNSRLSKRSFSADGFEMLQNPVKDSKEMFQTYKQMYLEKRSRSLGSSPVK- | Homo sapiens chromosome 12 open reading frame 35 (C12orf35), |
| 30 | NSSLRKKIKKKK- | no match |
| 31 | NSSIHYINFFQLSVFLHNSSKLKNCTSGQARWVTPVNLSTLGG- | non-coding region of the cDNA |
| 32 | NSSNKPAVTTKSPAVKPAAAPKQPVGGGQKLLTRKADSSSSEEESSSSEEEKTKKMVATTKPKATAKAALSLPAKQAPQGSRDSSSDSDSSSSEEEEEKTSKSAVKKKPQKVAGGAAPSKPASAKKGKAESSNSSSSDDSSEEEEEKLKGKGSPRPQAPKANGTSALTAQNGKAAKNSEEEEEEKKKAAVVVSKSGSLKKRKQNEAAKEAETPQAKKIKLAAALE- | Homo sapiens nucleolar and coiled-body phosphoprotein 1 (NOLC1), |
| 33 | NSDRTKTARKNRIKKVEKKKKQNKTWEV- | genome sequence |
| 34 | NSSPSGVWWHSPPRPMWTPKDLSLLCHLK- | non-coding region of the cDNA |
| 35 | NSYWVGEDSTYKFFEVILIDPFHKAIRRNPDTQWITKPVHKHREMRGLTSAGRKSRGLGKGHKFHHTIGGSRRAAWRRRNTLQLHRYR- | Homo sapiens ribosomal protein L15 (RPL15), |
| 36 | NSAICQPQ- | genome sequence |
| 38 | NSSNKPAVTTKSPAVKPAAAPKQPVGGGQKLLTRKADSSSSEEESSSSEEEKTKKMVATTKPKATAKAALSLPAKQAPQGSRDSSSDSDSSSSEEEEEKTSKSAVKKKPQKVAGGAAPSKPASAKKGKAESSNSSSSDDSSEEEEEKLKGKGSPRPQAPKANGTSALTAQNGKAAKNSEEEEEEKKKAAVVVSKSGSLKKRKQNEAAKEAETPQAKKIKLAAALE- | Homo sapiens nucleolar and coiled-body phosphoprotein 1 (NOLC1), |
| 39 | NSSNKPAVTTKSPAVKPAAAPKQPVGGGQKLLTRKADSSSSEEESSSSEEEKTKKMVATTKPKATAKAALSLPAKQAPQGSRDSSSDSDSSSSEEEEEKTSKSAVKKKPQKVAGGAAPSKPASAKKGKAESSNSSSSDDSSEEEEEKLKGKGSPRPQAPKANGTSALTAQNGKAAKNSEEEEEEKKKAAVVVSKSGSLKKRKQNEAAKEAETPQAKKIKLAAALE- | Homo sapiens nucleolar and coiled-body phosphoprotein 1 (NOLC1), |
| 40 | NSSIFCYDVCVESGCADIQLLLLCPTKKKKKKKKKKKKKKKKKKKKKKKKKGGGPPQKKKTPPGGPKRGGGGEKKGGGGARNFFFFFFFFFFFFFFTFLLIFFFFFFWEERKKKAGGAPPPPPPHQRKKKSKPVLPAELIDPRARRERGEEGRGSLHCVGLDNAHRRLEAERGNSSGRGGITHCNIRGILR- | genome sequence |
| 41 | NSSNKPAVTTKSPAVKPAAAPKQPVGGGQKLLTRKADSSSSEEESSSSEEEKTKKMVATTKPKATAKAALSLPAKQAPQGSRDSSSDSDSSSSEEEEEKTSKSAVKKKPQKVAGGAAPSKPASAKKGKAESSNSSSSDDSSEEEEEKLKGKGSPRPQAPKANGTSALTAQNGKAAKNSEEEEEEKKKAAVVVSKSGSLKKRKQNEAAKEAETPQAKKIKLAAALE- | Homo sapiens nucleolar and coiled-body phosphoprotein 1 (NOLC1), |
| 43 | NSSNKPAVTTKSPAVKPAAAPKQPVGGGQKLLTRKADSSSSEEESSSSEEEKTKKMVATTKPKATAKAALSLPAKQAPQGSRDSSSDSDSSSSEEEEEKTSKSAVKKKPQKVAGGAAPSKPASAKKGKAESSNSSSSDDSSEEEEEKLKGKGSPRPQAPKANGTSALTAQNGKAAKNSEEEEEEKKKAAVVVSKSGSLKKRKQNEAAKEAETPQAKKIKLAAALE- | Homo sapiens nucleolar and coiled-body phosphoprotein 1 (NOLC1), |
| 44 | NSS- | genome sequence |
| 45 | NSSNKPAVTTKSPAVKPAAAPKQPVGGGQKLLTRKADSSSSEEESSSSEEEKTKKMVATTKPKATAKAALSLPAKQAPQGSRDSSSDSDSSSSEEEEEKTSKSAVKKKPQKVAGGAAPSKPASAKKGKAESSNSSSSDDSSEEEEEKLKGKGSPRPQAPKANGTSALTAQNGKAAKNSEEEEEEKKKAAVVVSKSGSLKKRKQNEAAKEAETPQAKKIKLAAALE- | Homo sapiens nucleolar and coiled-body phosphoprotein 1 (NOLC1), |
| 47 | NSKYTINSKGSTPKHIIVKL- | genome sequence |
| 48 | NSAWHLPRMLTG- | genome sequence |
| 49 | NSAKSV- | no match |
| 50 | NSSNKPAVTTKSPAVKPAAAPKQPVGGGQKLLTRKADSSSSEEESSSSEEEKTKKMVATTKPKATAKAALSLPAKQAPQGSRDSSSDSDSSSSEEEEEKTSKSAVKKKPQKVAGGAAPSKPASAKKGKAESSNSSSSDDSSEEEEEKLKGKGSPRPQAPKANGTSALTAQNGKAAKNSEEEEEEKKKAAVVVSKSGSLKKRKQNEAAKEAETPQAKKIKLAAALE- | Homo sapiens nucleolar and coiled-body phosphoprotein 1 (NOLC1), |
| 51 | NSSNKPAVTTKSPAVKPAAAPKQPVGGGQKLLTRKADSSSSEEESSSSEEEKTKKMVATTKPKATAKAALSLPAKQAPQGSRDSSSDSDSSSSEEEEEKTSKSAVKKKPQKVAGGAAPSKPASAKKGKAESSNSSSSDDSSEEEEEKLKGKGSPRPQAPKANGTSALTAQNGKAAKNSEEEEEEKKKAAVVVSKSGSLKKRKQNEAAKEAETPQAKKIKLAAALE- | Homo sapiens nucleolar and coiled-body phosphoprotein 1 (NOLC1), |
| 52 | NSYWVGEDSTYKFFEVILIDPFHKAIRRNPDTQWITKPVHKHREMRGLTSAGRKSRGLGKGHKFHHTIGGSRRAAWRRRNTLQLHRYR- | Homo sapiens ribosomal protein L15 (RPL15), |
| 53 | NSSNKPAVTTKSPAVKPAAAPKQPVGGGQKLLTRKADSSSSEEESSSSEEEKTKKMVATTKPKATAKAALSLPAKQAPQGSRDSSSDSDSSSSEEEEEKTSKSAVKKKPQKVAGGAAPSKPASAKKGKAESSNSSSSDDSSEEEEEKLKGKGSPRPQAPKANGTSALTAQNGKAAKNSEEEEEEKKKAAVVVSKSGSLKKRKQNEAAKEAETPQAKKIKLAAALE- | Homo sapiens nucleolar and coiled-body phosphoprotein 1 (NOLC1), |
| 54 | NSSLTGVL- | genome sequence |
| 55 | NSAPRGDRRGCQLNLLQKWKRSRKRQQRRINLQTKKCKQKGKGEQRENRPKWLTKKLKKTYLRKTGKRRLRRVQPLMKQERKKPSLINNHIPCLISGPCLPSCTIQRNIFINYFVNASFLVALETFLRRRESHLIPFFKCKCFFLRGEIICWLFIFWYNQKLAAALE- | frameshift |
| 56 | NSSNKPAVTTKSPAVKPAAAPKQPVGGGQKLLTRKADSSSSEEESSSSEEEKTKKMVATTKPKATAKAALSLPAKQAPQGSRDSSSDSDSSSSEEEEEKTSKSAVKKKPQKVAGGAAPSKPASAKKGKAESSNSSSSDDSSEEEEEKLKGKGSPRPQAPKANGTSALTAQNGKAAKNSEEEEEEKKKAAVVVSKSGSLKKRKQNEAAKEAETPQAKKIKLAAALE- | Homo sapiens nucleolar and coiled-body phosphoprotein 1 (NOLC1), |
| 57 | NSSRTQRRSVIWFTQ- | genome sequence |
| 58 | NSAFII- | non-coding region of the cDNA |
| 59 | NSMRRSRSTRSWRRRRSVWPAAGRSSTHTS- | frameshift |
| 61 | NSARAPACAPSRARAMPSDRPFKQRRSFADRCKEVQQIRDQHPSKIPVIIERYKGEKQLPVLDKTKFLVPDHVNMSELVKIIRRRLQLNPTQAFFLLVNQHSMVSVSTPIADIYEQEKDEDGCLRPHSSN- | Homo sapiens microtubule-associated protein 1 light chain 3 alpha  (MAP1LC3A) |
| 62 | NSSISQLSPPSHLQ- | frameshift |
| 63 | NSAKSV- | non-coding region of the cDNA |
| 64 | NSSNKPAVTTKSPAVKPAAAPKQPVGGGQKLLTRKADSSSSEEESSSSEEEKTKKMVATTKPKATAKAALSLPAKQAPQGSRDSSSDSDSSSSEEEEEKTSKSAVKKKPQKVAGGAAPSKPASAKKGKAESSNSSSSDDSSEEEEEKLKGKGSPRPQAPKANGTSALTAQNGKAAKNSEEEEEEKKKAAVVVSKSGSLKKRKQNEAAKEAETPQAKKIKLAAALE- | Homo sapiens nucleolar and coiled-body phosphoprotein 1 (NOLC1), |

: initial parts of the sequence that originate from the T7 phage.
