# Supplementary material for: Screening E3 Substrates Using a Live Phage Display Library
Source: PLoS One. 2013 Oct 4;8(10):e76622. doi: 10.1371/journal.pone.0076622 (PMC3790729; doi:10.1371/journal.pone.0076622)
Supplement: Table S5 — Unnatural peptides identified at least twice in four independent screenings. +: positive result; -: nagetive result; NA: do not tested. (DOC) [file pone.0076622.s006.doc]

Table S5. Unnatural peptides identified at least twice in four independent screenings.

+: positive result; -: negative result; NA: do not tested.
