# Supplementary material for: Screening E3 Substrates Using a Live Phage Display Library
Source: PLoS One. 2013 Oct 4;8(10):e76622. doi: 10.1371/journal.pone.0076622 (PMC3790729; doi:10.1371/journal.pone.0076622)
Supplement: Table S6 — Quantification of exogenous ubiquitinated RPL36a using ImageJ in MDM2 overexpressed HEK293T cells. (DOC) [file pone.0076622.s007.doc]

Table S6. Quantification of exogenous ubiquitinated RPL36a using ImageJ in MDM2 overexpressed HEK293T cells

|  | IntDen | |  |  |
| --- | --- | --- | --- | --- |
|  | ubiquitin bands | GFP-RPL36a | Relative Poly-Ub-RPL36a | Standarized Relative Poly-Ub-RPL36a |
| - | 464032 | 222228 | 2.09 | 1.00 |
| MDM2Δring | 835137 | 188947 | 4.42 | 2.12 |
| MDM2WT | 2145215 | 333778 | 6.43 | 3.08 |

IntDen:Integrated Density
