# Supplementary material for: Screening E3 Substrates Using a Live Phage Display Library
Source: PLoS One. 2013 Oct 4;8(10):e76622. doi: 10.1371/journal.pone.0076622 (PMC3790729; doi:10.1371/journal.pone.0076622)
Supplement: Table S7 — Quantification of exogenous RPL36a using ImageJ in MDM2 overexpressed HEK293T cells. (DOC) [file pone.0076622.s008.doc]

Table S7. Quantification of exogenous RPL36a using ImageJ in MDM2 overexpressed HEK293T cells

|  | IntDen | |  |  |
| --- | --- | --- | --- | --- |
|  | RPL36a | beta-actin | relative IntDen | standarized relative IntDen |
| - | 185150 | 269876 | 0.69 | 1.00 |
| MDM2 | 116594 | 434370 | 0.27 | 0.39 |
| MDM2+MG132(10uM) | 372268 | 504081 | 0.74 | 1.08 |
| MDM2+MG132(20uM) | 599273 | 645646 | 0.93 | 1.35 |

IntDen:Integrated Density
